# Regional and local patterns of genetic variation and structure in yellow‐necked mice ‐ the roles of geographic distance, population abundance, and winter severity

**DOI:** 10.1002/ece3.4291

**Published:** 2018-07-22

**Authors:** Sylwia D. Czarnomska, Magdalena Niedziałkowska, Tomasz Borowik, Bogumiła Jędrzejewska

**Affiliations:** ^1^ Mammal Research Institute Polish Academy of Sciences Białowieża Poland; ^2^ Present address: Institute of Biochemistry and Biophysics of the Polish Academy of Sciences (IBB PAS) Warszawa Poland

**Keywords:** *Apodemus flavicollis*, genetic structure, January temperature, microsatellites, northeastern Poland, population density

## Abstract

The goal of this study, conducted in seven large woodlands and three areas with small woodlots in northeastern Poland in 2004–2008, was to infer genetic structure in yellow‐necked mouse *Apodemus flavicollis* population and to evaluate the roles of environmental and population ecology variables in shaping the spatial pattern of genetic variation using 768 samples genotyped at 13 microsatellite loci. Genetic variation was very high in all studied regions. The primal genetic subdivision was observed between the northern and the southern parts of the study area, which harbored two major clusters and the intermediate area of highly admixed individuals. The probability of assignment of individual mice to the northern cluster increased significantly with lower temperatures of January and July and declined in regions with higher proportion of deciduous and mixed forests. Despite the detected structure, genetic differentiation among regions was very low. Fine‐scale structure was shaped by the population density, whereas higher level structure was mainly shaped by geographic distance. Genetic similarity indices were highly influenced by mouse abundance (which positively correlated with the share of deciduous forests in the studied regions) and exhibited the greatest change between 0 and 1 km in the forests, 0 and 5 km in small woodlots. Isolation by distance pattern, calculated among regions, was highly significant but such relationship between genetic and geographic distance was much weaker, and held the linearity at very fine scale (~1.5 km), when analyses were conducted at individual level.

## 1 INTRODUCTION

One of the key concepts of population genetics is isolation by distance (IBD), where the neutral genetic variation is shaped mostly by limited dispersal of individuals, and genetic differentiation is linearly correlated with geographic distance and cumulates over generations (Wright, 1943). However, the model is based on the unrealistic assumptions that populations are large, equal in size, and stable over time. Species that undergo cyclic density fluctuations are extreme cases of demographic instability. Computer simulations showed that IBD pattern depends strongly on how populations differ in size and how they fluctuate, but it does not depend on the gene flow frequency or pattern (Björklund, Bergek, Ranta, & Kaitala, 2010). In the cyclic species which undergo demographic fluctuations over shorter periods of time (i.e., less than 10 generations), the spatial genetic structure at a particular time may be shaped by both present‐time and recent‐past demographic events. During the phase of low density, genetic drift is expected to reduce genetic diversity and substantially increase genetic differentiation among isolated subpopulations (Leblois, Rousset, & Estoup, 2004). In a low phase of population fluctuations, spatial aggregations of small mammals form temporary metapopulation structures (e.g., Lima, Marquet, & Jaksic, 1996). Dispersal of individuals among suitable patches of habitat is an essential ingredient of metapopulation dynamics. It was also reported by Szacki and Liro (1991) and Szacki, Babińska‐Werka, and Liro (1993) that rodents move longer distances in heterogeneous forest habitat and suburban habitat compared with more homogeneous forest. Increased mobility was suggested as a behavioral adaptation to fragmented landscape.

Previous studies on bank voles *Clethrionomys glareolus* (Borkowska, 1999; Gębczyński & Ratkiewicz, 1998) and yellow‐necked mouse *Apodemus flavicollis* (Wójcik, 1993), which analyzed genetic variation using protein electrophoresis, suggested that density fluctuations might cause serious genetic bottlenecks in periods of low population densities. However, several microsatellite DNA studies conducted during low phases of population density (Aars et al., 2006; Berthier, Charbonnel, Galan, Chaval, & Cosson, 2006; Pilot, Dąbrowski, Jancewicz, Schtickzelle, & Gliwicz, 2010; Rikalainen, Aspi, Galarza, Koskela, & Mappes, 2012) did not detect any substantial loss of genetic variability in metapopulations. Studies conducted by Ehrich, Yoccoz, and Ims (2009) on two numerically dominant vole species of northern Fennoscandia, red voles *Clethrionomys rutilus,* and gray‐sided voles *C. rufocanus,* showed that high‐amplitude density fluctuations increased gene flow and genetic variability in vole population. It was also noted that spring densities, when genetic bottleneck could be observed, had no effect on differentiation or local genetic diversity. In certain cases, subdivided population with frequent local extinctions may retain more genetic diversity because founders of newly colonized patches tend to be a mixture from different subpopulations rather than from one larger source population, which increase effective population size (*N*_e_). The fraction of genetic variation lost during the bottleneck is a function of the population growth rate (Nei, 1975). Populations, which recover quickly after the bottleneck, lose little genetic variation even after severe density crash. Moreover, overlapping generations can minimize the effect of environmental fluctuations on population size and thus on the level of maintained genetic variability (Gaggiotti, 2003). Adult individuals reproduce several times throughout their lives; therefore, the genetic variation observed in a given cohort is more likely to be transferred to future generations than in case of organisms with discrete generations (Ray, 2001).

Genetic variability level and its spatial pattern found in rodent populations can be relatively complex because of interactions between landscape, geography, and demography (Kaitala, Ranta, & Stenseth, 2006). Spatial genetic structure may result from a general isolation‐by‐distance pattern locally distorted by particular landscape structures (Arnaud, 2003; Vos, Antonisse‐De Jong, Goedhart, & Smulders, 2001). Several empirical studies have shown the effect of ecological barriers or landscape characteristics on population genetic structure at different spatial scales (Angelone, Kienast, & Holderegger, 2011; Pope, Domingo‐Roura, Erven, & Burke, 2006). Wide highways, rivers, and other linear habitats restrict the movement and gene flow in small animals (Aars, Ims, Liu, Mulvey, & Smith, 1998; Gerlach & Musolf, 2000; Keller, Nentwig, & Largiadèr, 2004; Rico, Kindlmann, & Sedláček, 2007; Shepard, Kuhns, Dreslik, & Phillips, 2008). There is an ongoing discussion whether the local substructuring observed in several small mammal species is genuinely determined by landscape heterogeneity and low level of effective small‐scale dispersal between isolated subpopulations or by social structure and behavior (e.g., Gerlach & Musolf, 2000; Lougheed, Gibbs, Prior, & Weatherhead, 1999; Mossman & Waser, 2001; Redeker et al., 2006; Schweizer, Excoffier, & Heckel, 2007). In addition, the isolation‐by‐distance pattern varies between areas of different vole abundance (Berthier, Galan, Foltete, Charbonnel, & Cosson, 2005). Dispersal pattern changes over time throughout the demographic cycle (Gauffre et al., 2014) and density‐dependent dispersal are observed in most abundant rodent species (Kozakiewicz, Gortat, Kozakiewicz, & Barkowska, 1999; Stenseth, Viljugrein, Jędrzejewski, Mysterud, & Pucek, 2002).

**Figure 1:**
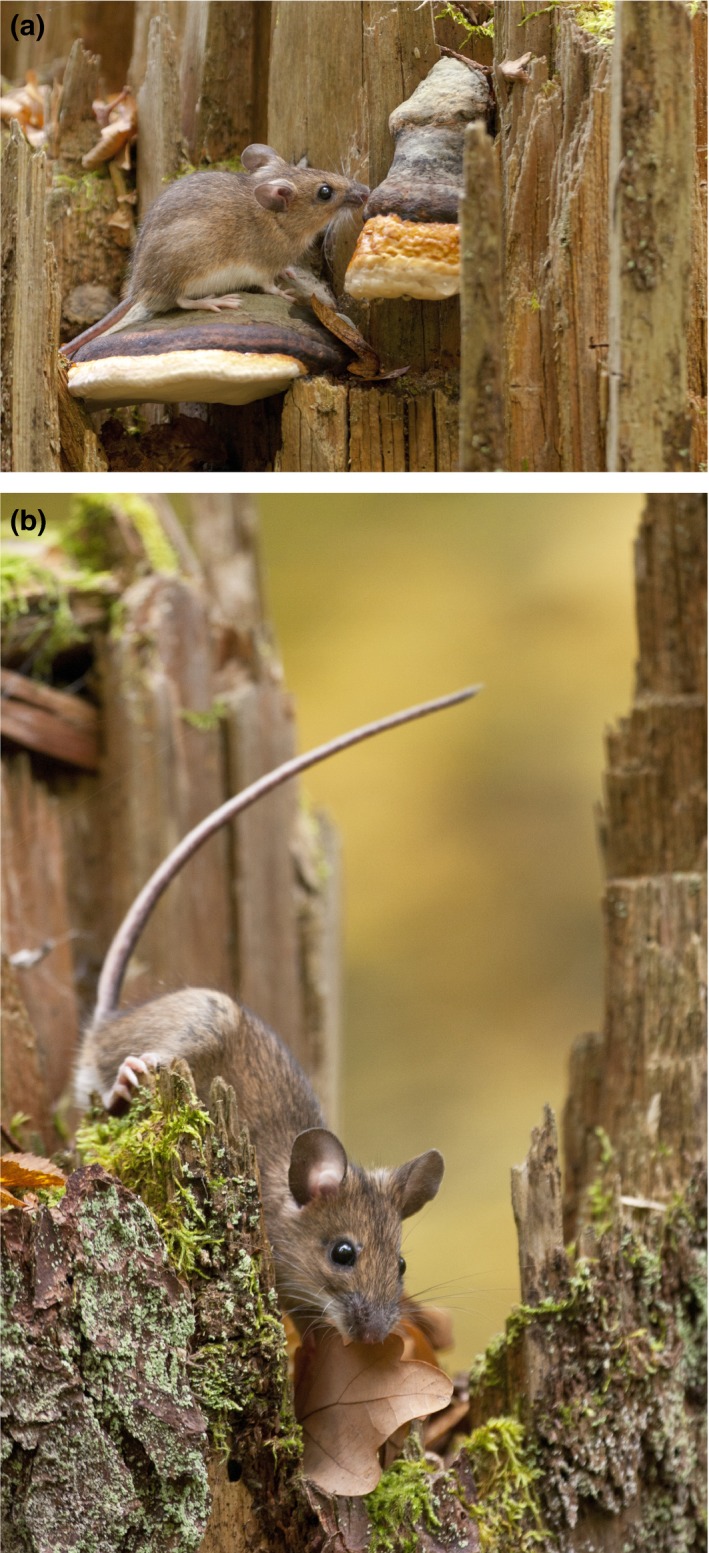
Yellow‐necked mouse *Apodemus flavicollis* is one of the most abundant rodent species in forests of northeastern Poland. (Photo: Karol Zub)

Yellow‐necked mouse (Figure 1) is one of the most numerous forest rodent species in Central European temperate forest zone (Niedziałkowska, Kończak, Czarnomska, & Jędrzejewska, 2010). It prefers deciduous forest (especially oak–lime–hornbeam stands) and generally feeds on tree seeds with slight supplementation of its diet with invertebrates (Butet & Delettre, 2011; Gębczyńska, 1976; Hansson, 1985). Recent isotope analyses of rodent have shown that yellow‐necked mice are strongly associated with mast of deciduous trees (>80% of diet; Selva, Hobson, Cortés‐Avizanda, Zalewski, & Donázar, 2012). Population dynamics of yellow‐necked mice is largely shaped by seed crops of deciduous trees. In eastern Poland, heavy crops (usually synchronous in common oak *Quercus robur*, hornbeam *Carpinus betulus*, and maple *Acer platanoides*) occur at 6‐ to 9‐year intervals and trigger winter breeding in mice, their outbreak in the following year, and crash in the next year (Pucek, Jędrzejewski, Jędrzejewska, & Pucek, 1993). Two years of outbreak‐crash are followed by 4–7 years of moderate densities with winter mortality averaging 86% of autumn numbers of mice (Pucek et al., 1993).

European *Apodemus* sp. are thought to be habitat generalists with high colonization potential in heterogeneous habitat (Liro & Szacki, 1995; Wegner & Henein, 1991). Movements longer than 1,000 m have been observed for *Apodemus agrarius* (Szacki & Liro, 1991) and *A. sylvaticus* (Geuse, Bauchau, & LeBoulenge, 1985; Wolton, 1985). Home ranges of yellow‐necked mice varied from 100 to 3,950 m^2^ depending on population density and social status of the individual (Matič, Vukicevic‐Radic, Stamenkovic, & Kataranovski, 2007).

Genetic studies on yellow‐necked mice are scarce and mostly restricted to phylogeography (Michaux, Libois, & Filippucci, 2005; Michaux, Libois, Paradis, & Filippucci, 2004). The only studies that aimed at including landscape features in explaining the genetic structure of yellow‐necked mice had been focused on evaluating the direct effect of natural and anthropogenic barriers on defined a priori population and covered a small geographical scale (Gortat et al., 2010; Kozakiewicz et al., 2009; Rico, Kindlmann, & Sedláček, 2009).

The aims of this study were to infer genetic structure in yellow‐necked mice (Figure 1) population and to evaluate the role of environmental and population ecology variables in shaping the observed pattern of genetic pattern of the species in northeastern Poland. To address these questions, we combined microsatellite, ecological (population density and habitat structure in study sites), and environmental (geographic distance, climatic conditions) data. Given the ecological characteristic of the species, we were particularly interested in testing the hypotheses that: (a) yellow‐necked mouse population is characterized by high level of genetic variation structured in a complex hierarchical way, (b) mouse abundance has profound effect on local population structure, and (c) genetic structure is related to ecological and environmental conditions in the study sites.

### 1.1 Study area

**Figure 2:**
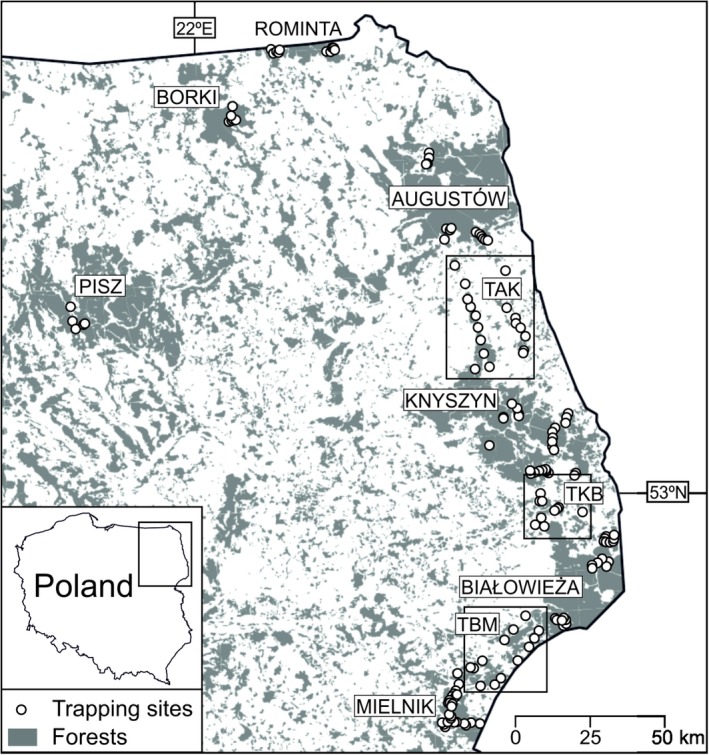
The study area and trapping sites for the yellow‐necked mouse in NE Poland. Augustów (AUG), Białowieża (BIAL), Borki (BOR), Knyszyn (KNYSZ), Mielnik (MIEL), Pisz (PIS) and Rominta (ROM) forests as well as three transects (T) among four of them: Augustów–Knyszyn (TAK), Knyszyn–Białowieża (TKB), Białowieża–Mielnik transects (TBM)

The study was conducted in seven large woodlands located in the lowlands of northeastern Poland: Augustów (AUG), Białowieża (BIAL), Borki (BOR), Knyszyn (KNYSZ), Mielnik (MIEL), Pisz (PISZ) and Rominta (ROM) Forests, as well as in more open terrain with small, fragmented woodlots along three transects (T) among four of the above listed woodlands: Augustów–Knyszyn (TAK), Knyszyn–Białowieża (TKB), Białowieża–Mielnik (TBM). The study area (53°56′–54°36′ N, 21°04′–23°94′ E) spanned a maximum distance of 230 km in the S–N direction and 180 km in the E–W (Figure 2). The landscape of the region has been shaped by glaciations (mainly the Riss, 310,000 to 130,000 yBP, and the Würm, 70,000 to 10,000 yBP). It is mainly a plain, with some belts of frontal and moraine hills and numerous postglacial lakes. The altitude is between 25 and 312 m a.s.l. Forest cover is slightly higher (29.9%) than the mean for the country (28.7%) (Statistical Yearbook of the Republic of Poland, 2005). The climate is transitional between continental and Atlantic types. Annual precipitation is 550–700 mm. Snow cover lasts for 76–90 days, and the growing season lasts for 180–200 days (Kondracki, 1972). During the study period, the mean temperature in January was −7°C and that in July 22°C (Supporting Information Table S1).

Most of the sampling sites were located in managed forests administrated by the Olsztyn and Białystok Regional Forest Directorates. Minor parts of Białowieża and Augustów Forests are protected as national parks, and from two to 24 small nature reserves are located in each of the seven woodlands. The dominant tree species were Scots pine (*Pinus silvestris*), Norway spruce (*Picea abies*), common oak, silver birch (*Betula pendula*), white birch (*B. pubescens*), and common alder (*Alnus glutinosa*). The woodlands differed in size (from 126 to 1,600 km^2^), productivity, and mean age of tree stands. The Białowieża Forest is the oldest and the best preserved woodland in the whole study area (Sokołowski, 2006). Small woodlots along transects represented significantly younger and often highly fragmented tree stands. Environmental conditions varied among the studied regions (Supporting Information Table S1). Wooded areas constituted 83.6%–98.8% in forests (mean forest cover 90.4%) and 54.9%–69.7% along transects (mean 62.2%). The highest share of deciduous forest was found in Mielnik Forest (68.6% of the forested area) and Augustów‐Knyszyn Transect (46.3% of the forested area).

## 2 MATERIAL AND METHODS

### 2.1 Trapping of mice

Yellow‐necked mice (Figure 1) were live‐trapped in the seven woodlands (PIS, BOR, ROM, AUG, KNYSZ, BIAL, MIEL) from mid June until early September in 2004–2006 and in four woodlands (AUG, KNYSZ, BIAL, MIEL) and along three transects (TAK, TKB, TBM) from early July to late September in 2007–2008. Each year, animals were captured in different part of the woodland or transect, with sequence of surveying that allowed for trapping in the same woodland or transect in different months of the season. We set traps in eight to 11 new sites spanning 5 to 20 km in the woodlands and 4 to 42 km along the transects. None of the circular trapping sites was resampled during the study.

At each site, two types of livetraps were placed: small wooden traps (Dziekanów type, 16.5 × 9.5 × 8 cm) for rodents, large wooden boxes (11 × 11 × 24.5 cm) for dormice (applicable also for mice). Six to 10 small traps were placed on the ground, five large boxes were located on shelves stuck by sticky tape to tree trunks at a height of about 2 m. The trapping and sampling was conducted according to the protocol described by Niedziałkowska et al. (2010). In total, 820 individuals of yellow‐necked mouse were captured at 176 trapping sites within a period of 2004–2008. Tissue samples for DNA analysis (tail or ear clipping) were placed in a tube with 96% alcohol and stored in −20°C upon DNA extraction. All capture procedures were in accordance with Polish law and were accepted by the Local Ethical Commission in Białystok (permissions nos 07/2004, 15/2006, 65/2007). Trapping site locations were approved by the administrators of the area. All animals were released at the place of capturing.

### 2.2 Habitat structure and climatic data of trapping sites as analyzed with GIS tools

Precise location of each trapping site was identified using GPS GARMIN60CSx. Complete botanical description of forest floor vegetation, undergrowth and tree stand was collected for each trapping site. Based on this description, each plot was classified according to forest habitat type as coniferous, mixed or deciduous forest. In program ArcGIS (ESRI), a buffer was defined around each trapping site with a radius equal to the mean distance between all trapping sites (~1 km). Next, Corine Landcover 2006 (CLC2006) maps were used to measure the percentage cover of each land use category (coniferous, mixed and deciduous forests, meadows and arable lands, waters and other) within a defined buffer zone (Supporting Information Table S1). Moderate Resolution Imaging Spectroradiometer (MODIS) was used to collect land surface temperature (LST, Wan, Zhang, Zhang, & Li, 2003) with high spatial (grid 1 × 1 km) and temporal (four measurements per month for each trapping plot for 2004–2008) resolution. Collinearity between the explanatory variables was assessed by calculating pairwise Pearson's correlation coefficients and only uncorrelated variables (*R* < |0.5|) were selected for further steps of analysis: mean temperature of January (*T*_JAN_), mean temperature of July (*T*_JUL_), and share of deciduous‐mixed forest (DMF, the most suitable habitat for yellow‐necked mouse) in 1‐km buffer zone around the trapping sites.

### 2.3 Laboratory analysis

Genomic DNA was extracted using DNeasy Blood & Tissue Kit (QIAGEN) according to the manufacturer's protocol. Quantity and quality control was performed using spectrophotometer NanoDrop ND‐1000. We selected 15 markers that were tested previously on yellow‐necked mouse by other researchers and therefore guaranteed amplification success: AF246520, AF246522, AF246523 (Harr, Musolf, & Gerlach, 2000), MSAf‐3, MSAf‐7, MSAf‐8, MSAf‐16, MSAf‐22 (Gockel et al., 1997), CAA2A, GTTA1A, GTTC4A, GTTD8S, GACAD1A, GCATD7S, TNF (Makova, Patton, Krysanov, Chesser, & Baker, 1998). PCR mixture consisted of 2.5 μl of HotStart Taq Master Mix Kit (QIAGEN), 0.4 μl of each 25pM primer (forward primer fluorescently labeled), 1.85 μl of purified water and 0.5 μl of DNA. PCR reaction of AF246520, AF246522, AF246523, MSAf‐3, MSAf‐7, MSAf‐8, MSAf‐16, MSAf‐22 was performed in 30 cycles (30 s at 94°C, 30 s at 54°C and 1 min at 72°C) with initial denaturation for 15 min at 94°C and final elongation for 10 min at 72°C. PCR reaction of CAA2A, GTTA1A, GTTC4A, GTTD8S, GACAD1A, GCATD7S, TNF was performed in 35 cycles of denaturation at 94°C for 60 s, annealing at 54°C, 58°C or 60°C for 30 s (following the protocol in Makova et al., 1998) and extension at 72°C for 60 s with initial step of denaturation for 15 min at 94°C and final elongation for 3 min at 72°C. A mixture containing 1 μl of diluted PCR product, 0.2 μl of GeneScan 400 ROX size standard (Applied Biosystem) and 10 μl of Hi–Di Formamide (Applied Biosystem) was separated on Applied Biosystems 3100 DNA Analyser. Genotypes were determined with GeneMarker ver. 1.75 (SoftGenetics LLC, 2007). All PCR reactions were carried out using a DNA Engine Dyad Peltier Thermal Cycler (BIO RAD).

A final data set consisted of 768 samples genotyped at 13 microsatellite loci (GTTA1A and GTTD8S were excluded due to poor amplification and detected monomorphism). Detailed information on the number of analyzed samples from each woodland, transect, and year is presented in Supporting Information Table S2.

### 2.4 Statistical analysis of genetic data

Microsatellite variability statistics, calculated per locus and per geographic region (number of alleles per locus, number of private alleles, observed and expected heterozygosity) were calculated in GenAlEx (Peakall & Smouse, 2006). Allelic richness and inbreeding coefficient (*F*_IS_) were calculated using FSTAT (Goudet, 1995). Micro‐Checker 2.2.3 (Van Oosterhout, Hutchinson, Wills, & Shipley, 2004) was used to check the data for putative null alleles and scoring errors. Genetic differentiation between geographic regions *F*_ST_ (Weir & Cockerham, 1984) was calculated in Arlequin ver. 3.11 (Excoffier, Laval, & Schneider, 2005). Mean values of Queller and Goodnight relatedness coefficient in each woodland and the transect were calculated in GenAlEx. We estimated effective population size *N*_e_ using a bias‐corrected version of the linkage disequilibrium method of Waples and Do (2008) with the option of random mating selected using NeEstimator V2.1 (Do et al., 2014).

STRUCTURE 2.3.4 was used to infer population structure and assign individuals to subpopulations (clusters) based on individual multilocus genotypes without any prior spatial information (Pritchard, Stephens, & Donnelly, 2000). STRUCTURE was run using 50^5^ MCMC iterations, following a burn‐in period of 50^4^ iterations with admixture model and correlated allele frequencies (Falush, Stephens, & Pritchard, 2003). Number of genetic groups (*K*) was tested from 1 to 10 with 10 runs completed per each *K*. It is also known that sampling scheme might significantly affect the assessment of population subdivision (Oyler‐McCance, Fedy, & Landguth, 2013; Schwartz & McKelvey, 2009). Therefore, additionally to the whole data set, we ran STRUCTURE analyses solely for mice collected in seven woodlands with model settings of no admixture (it assumes that each individual belongs to a single cluster, data not shown) and admixture (each individual draws some fraction of its genome from each of the *K* populations) and separately on samples collected in each year to check for the consistency of results among years. Program STRUCTURE HARVESTER v. 06.94 (Earl & Vonholdt, 2012) was used to summarize the results and produce the graph presenting the posterior probability of the data [Ln*P*(*D*)] and delta *K* (Evanno, Regnaut, & Goudet, 2005). Subsequently, Principal Component Analyses (PCA) were performed on individual genotypes from 10 geographical regions using the adegenet package (Jombart, 2008) in R (R Development Core Team, 2016). The PCA does not assume Hardy–Weinberg equilibrium (HWE) and is highly efficient in revealing genetic structure in the form of clines, which is more difficult to detect than clusters (Jombart, Pontier, & Durfour, 2009).

We also evaluated the genetic structure results by applying spatially explicit algorithms GENELAND ver. 3.3 (Guillot, Mortier, & Estoup, 2005) and TESS ver. 2.3.1 (Chen, Durand, Forbes, & Francois, 2007), Bayesian clustering programs that incorporate spatial coordinates into analyses. Additionaly, TESS allows to include “dummy points” when samples are not evenly distributed. Such points are not included in the analysis but help to avoid ghost populations. Inferences in GENELAND were performed in a single step as recommended by the author (Guillot, 2008). This approach makes inferences faster and avoids the issue of ghost populations. Thus, the MCMC were ran 50 times with settings as follows: delta.coord 0.05, 200,000 iterations, thinning 200, uncorrelated allele frequency, and 1–15 population. TESS was run using the MCMC algorithm with the admixture model. At first we used three initial starting values for the interaction parameter, which represents spatial interactions (*w* = 0.6, 0.75 and 0.9) to select the optimal interaction parameter using likelihood (*w* = 0.6). Next, 10 independent simulations were performed for each *K* value (2–12) using 50,000 iterations and a burn‐in period of 10,000 iterations to identify, which *K* values produced the highest likelihood runs (*K*_max_). Finally, we conducted 100 simulations for each *K*_max_ and calculated membership probabilities from the 20 highest likelihood simulations for each *K*_max_ (Chen et al., 2007).

To test for genetic differentiation between defined spatial groups, pairwise *F*_ST_ was calculated in Arlequin ver. 3.11 (Excoffier et al., 2005). The levels of significance for multiple tests were adjusted by the sequential Bonferroni method (*k* = 6) (Rice, 1989).

### 2.5 Association of genetic variation with ecological factors

Isolation by distance was assessed using linear Mantel test on defined regions and individual level in R package vegan (Oksanen et al., 2011). First, *F*_ST_ coefficient values calculated among 10 geographic regions were plotted against the mean geographic distances. Secondly, Rousset's genetic distances a_r_ (Rousset, 2000) were related to geographic distance matrices generated between 768 individuals in program SPAGeDi v. 1.2 (Hardy & Vekemans, 2002). Significance was determined using 999 permutations.

Fine‐scale genetic structure and dispersal distance were analyzed using spatial autocorrelation methods implemented in GenAlEx, performed separately for individuals from each region (woodland or transect). Pairwise genetic distance and geographical distance matrices were used to calculate autocorrelation coefficient (*r*) for each distance class presented as a correlogram. The geographical distances were calculated as Euclidean distances between samples and the analyses were performed with an equal distance classes set at 1 km. Numbers of the calculated distance classes differed between regions due to differences in the spatial distribution of individuals in each region. For each analysis, we used 999 permutations to test the hypothesis of no spatial genetic structure, and 1,000 bootstraps to estimate 95% confidence intervals for r in a given geographical distance (Peakall, Ruibal, & Lindenmayer, 2003).

The number of individuals captured in a trapping point per 100 trapnights was used as an index of mouse abundance. Mean values per woodland and transect were calculated. Dependence of mouse abundance as well as calculated N_e_ on optimal habitat share (deciduous forest) was tested. Moreover, relationship between positive autocorrelation coefficient and mouse abundance was calculated.

Mean values of genetic similarity/differentiation (Queller and Goodnight relatedness coefficient, Rousset's genetic distance) were calculated in SPAGeDi for six geographical distance classes. In case of individuals captured in the woodlands, the classes were defined as follows (in kilometers): <0.001, 0.001–1, 1.001–2, 2.001–3, 3.001–4, 4.001–5, >5, and for individuals trapped in woodlots on transects: <0.001, 0.001–5, 5.001–10, 10.001–15, 15.001−20, 20.001–25, >25. Next, genetic parameters calculated for the first three distance classes in woodlands were plotted against the estimated abundance of rodents. Data from transects were not presented in that way due to small sample size (*n* = 3 transects).

Subsequently, we evaluated the association between environmental variables (mean temperature of January, mean temperature of July, and percentage cover of deciduous‐mixed forests in 1‐km buffer zone around trapping sites) and genetic variation measured as probability of each individual (*n* = 752) to be assigned to Cluster 1 (based on STRUCTURE with *K* = 2, see Section 2.1). We applied the generalized linear mixed models (GLMM) for binomial data using the “glmer” function implemented in the lme4 package (Bates, Maechler, Bolker, & Walker, 2015). We used mixed‐model framework with observation‐level random effect because our data expressed excess variation which had led to overdispersion problem. An information theoretic approach with a second‐order correction for small sample size (AIC_c_) (Burnham & Anderson, 2002) implemented in R package MuMIn (Bartoń, 2015) was applied to select the most parsimonious model, which best explained the observed spatial pattern in genetic variation of mice. Statistical analyses were performed in R (R Development Core Team, 2016).

## 3 RESULTS

### 3.1 Genetic variability and structure of the yellow‐necked mouse population

All 13 loci included in statistical analyses were highly polymorphic with number of alleles per locus ranging from 15 to 32 in the whole population of yellow‐necked mice in northeastern Poland (Supporting Information Table S3). Allelic richness, a parameter that controls for differences in a sample size, was similarly high in all regions ranging from 9.38 in Pisz Forest to 10.55 in Białowieża–Mielnik Transect (Table 1). Observed heterozygosity (*H*_o_) calculated per geographical region ranged from 0.776 to 0.842 and was lower than expected heterozygosity (*H*_e_) with values ranging from 0.841 to 0.869. All loci significantly deviated from Hardy–Weinberg equilibrium (HWE), when calculated for the entire population, but substantially lower number of loci showed lack of HWE when calculated per geographical region, with the exception of Knyszyn and Białowieża Forests, where consistently 10 and 11 loci significantly deviated from HWE. Proportion of detected private alleles was highest in Rominta and Białowieża Forests (0.69). In the whole sample of 10 regions, the proportion of private alleles was not correlated with the number of analyzed samples (*r* = 0.62, *p* = 0.057). The inbreeding coefficient values (*F*_IS_) were relatively small, with the highest values found in Knyszyn (0.108) and Białowieża Forests (0.101), and were correlated neither with samples size (*r* = 0.56, *p* = 0.091) nor with the mean pairwise Queller and Goodnight kinship coefficient (*r* = −0.54, *p* = 0.107).

**Table 1:** Basic genetic variability parameters for yellow‐necked mice from 10 geographical regions in NE Poland genotyped at 13 microsatellite loci. See Figure 2 for the names and location of the sampling regions. Sample sizes are shown in Supporting Information Table S2. *N*_A_, number of different alleles per locus; *N*_A_ >5%, number of different alleles per locus with frequency higher than 5%; *H*_O_, observed heterozygosity; *H*_E_, expected heterozygosity; *F*_IS_, inbreeding coefficient

Parameter	ROM	BOR	PIS	AUG	TAK	KNYSZ	TKB	BIAŁ	TBM	MIEL
*N*_A_	14.385	13.923	9.538	12.385	14.615	15.077	13.923	16.692	14.769	16.692
*N*_A_ > 5%	6.769	6.385	6.923	6.385	6.615	7.462	6.923	6.923	6.846	7.077
Allelic richness	10.260	9.968	9.380	9.475	9.674	10.341	9.885	10.172	10.552	10.386
No. private alleles	0.692	0.154	0.077	0.154	0.538	0.077	0.077	0.692	0.308	0.462
*H*_O_	0.842	0.791	0.809	0.816	0.803	0.786	0.818	0.776	0.797	0.794
*H*_E_	0.868	0.857	0.841	0.851	0.865	0.874	0.861	0.860	0.869	0.877
*F*_IS_	0.039	0.086	0.073	0.054	0.077	0.108	0.060	0.101	0.092	0.097

Genetic distances between geographical regions (calculated as *F*_ST_ coefficient) showed low level of differentiation with the highest values found between Pisz Forest and the rest of the regions (0.036–0.049, on average 0.039) and the lowest values among five southernmost areas: Knyszyn, Białowieża and Mielnik Forests and two transects: TKB, TBM (0.009–0.020, mean 0.012) (Table 2). All values of *F*_ST_ were highly significant.

**Table 2:** Pairwise genetic differentiation *F*_ST_ among yellow‐necked mice in 10 geographical regions (see Figure 2). All values of *F*_ST_ were statistically significant at *p* < 0.001 after Bonferroni correction

Geographical region	ROM	BOR	PIS	AUG	TAK	KNYSZ	TKB	BIAŁ	TBM
BOR	0.015								
PIS	0.031	0.032							
AUG	0.019	0.024	0.037						
TAK	0.024	0.028	0.040	0.014					
KNYSZ	0.023	0.030	0.038	0.020	0.009				
TKB	0.034	0.041	0.049	0.030	0.022	0.008			
BIAŁ	0.032	0.037	0.047	0.031	0.024	0.011	0.007		
TBM	0.031	0.033	0.036	0.027	0.022	0.016	0.014	0.007	
MIEL	0.027	0.035	0.041	0.028	0.022	0.017	0.020	0.014	0.009

**Figure 3:**
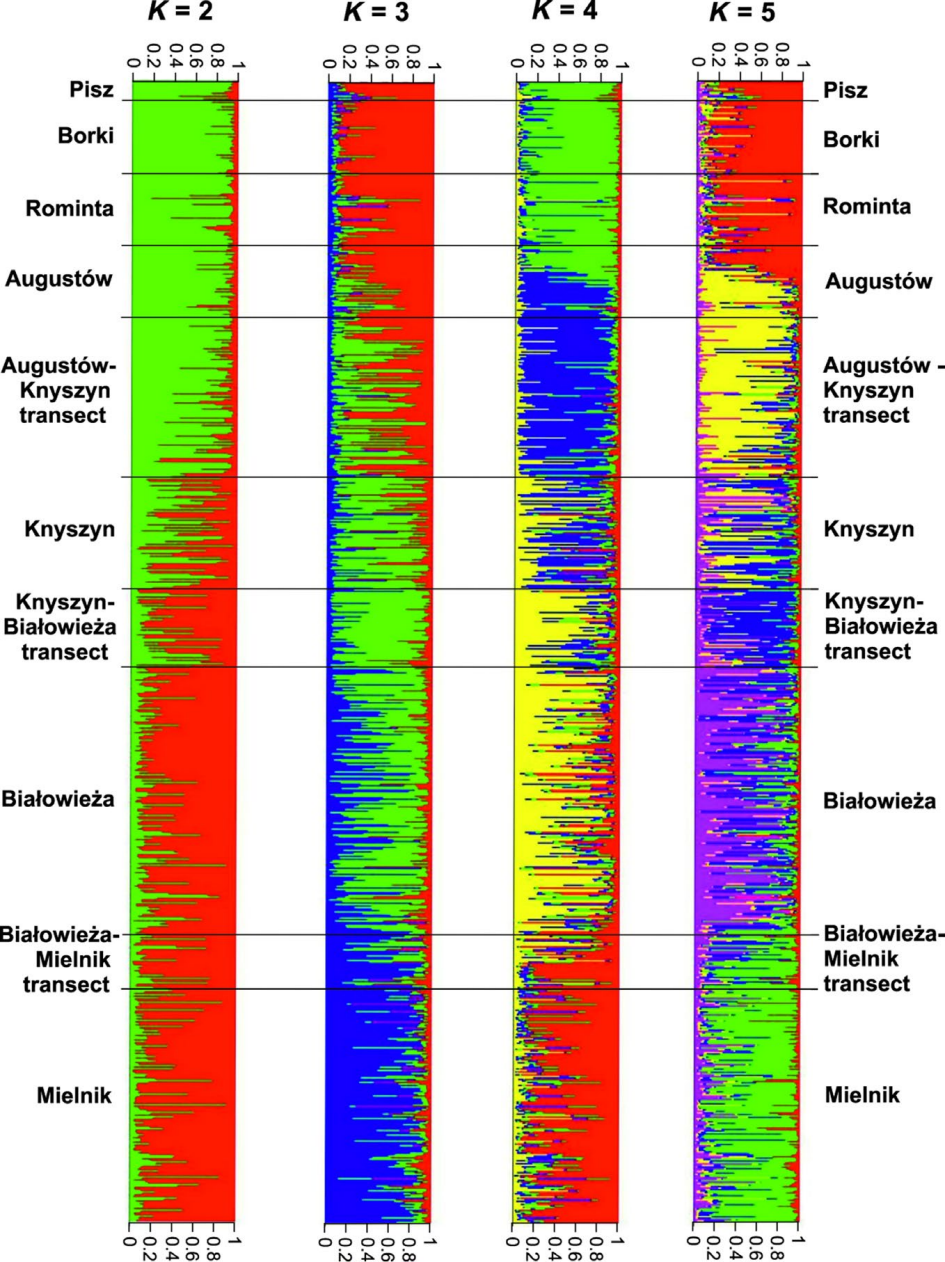
Results of STRUCTURE analysis of yellow‐necked mice based on 13 microsatellite markers assuming *K* = 2 to 5 genetic clusters. Black lines separate mice from different geographical regions. In *K* = 2 green color marks Cluster 1

Inferences in program STRUCTURE indicated major division of the yellow‐necked mouse population into two genetic clusters followed by reasonable divisions up to *K* = 5 (Figure 3). Although Ln*P*(*D*) continued to increase until it reached a plateau at *K* = 5, Δ*K* (Evanno et al., 2005) showed highest support for *K* = 2 with a slight increase at *K* = 4 and *K* = 5 (Supporting Information Table S4). Subdivision at *K* = 2 was characterized by genetic gradient from north to south, with Cluster 1 (green color in Figure 3), highly admixed individuals (assigned to both defined clusters) in Knyszyn Forest and Knyszyn–Białowieża Transect and Cluster 2 (red).

At K = 4, STRUCTURE identified the following subpopulations (Figure 3): (a) Pisz, Borki, Rominta and northern part of Augustów Forests, (b) southern Augustów Forest and Augustów–Knyszyn Transect, (c) Knyszyn–Białowieża Transect and Białowieża Forest, (d) Białowieża–Mielnik Transect and Mielnik Forest. Knyszyn Forest showed high level of admixture with adjacent subpopulations. With *K* = 5 the pattern was very similar, the only difference compared to *K* = 4 being some degree of separation of Knyszyn–Białowieża Transect from the neighboring forests (Figure 3).

Results of analyses in the Bayesian clustering programmes of mice captured only in seven woodlands confirmed the previously detected structure (data not shown) and indicated that analyses of samples collected along transects complemented the findings and did not change them significantly. The primal division between northern and southern areas, localized in the southern part of Augustów Forest, was highly concordant with the former results obtained for the whole data set. Analyses performed without Augustów–Knyszyn Transect indicated a sharper distinction between northern and southern parts of the study area, whereas inclusion of samples from the transect resulted in a more subtle genetic gradient. Importantly, spatial structuring observed in the yellow‐necked mouse population in NE Poland was also consistent between the consecutive years. High admixture of individuals from Knyszyn Forest was found also when analyses in STRUCTURE were performed solely on woodlands’ data with mice from transects excluded (data not shown).

**Figure 4:**
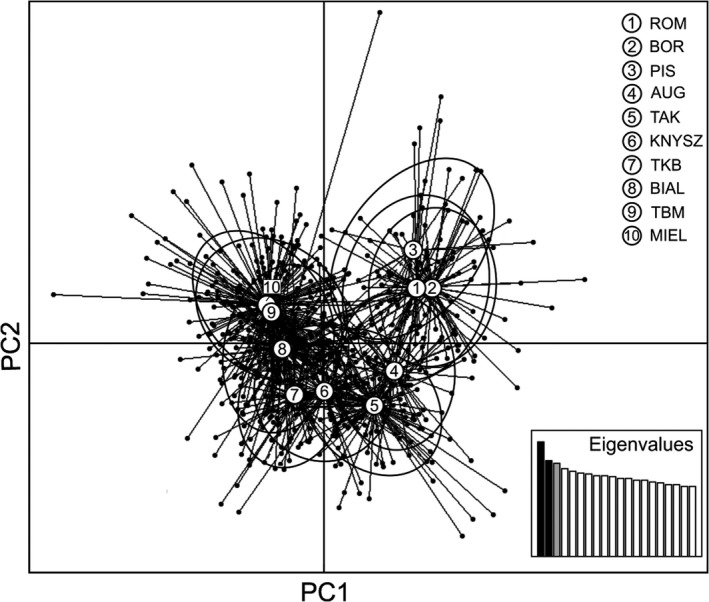
Principal Component Analysis (PCA) of yellow‐necked mice representing 10 geographic regions (see Figure 2). Black ovals are 95% inertia ellipses

Principal Component Analysis (PCA) revealed a major division of mice into two genetic groups visible on the first PC axis with individuals from northern part of the study area (Pisz, Borki and Rominta Forests) forming the most distinct cluster, and with further more subtle substructuring within the remaining samples (Figure 4). Mice from Augustów Forest and Augustów–Knyszyn Transect appeared to have an intermediate position between northern and southern regions according to the first PC axis. A gradient of genetic profiles within samples from Knyszyn Forest and the remaining regions was visible on the second axis and was in high concordance with the geographic distances.

**Figure 5:**
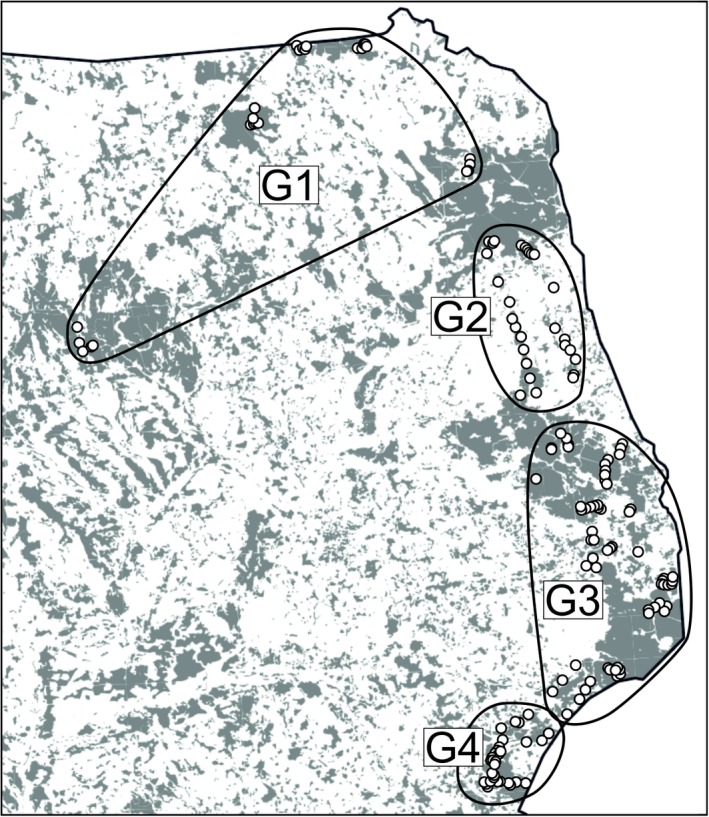
Structuring of yellow‐necked mouse population inferred by GENELAND in 50 runs. Individuals were assigned to four spatial groups in 41 of 50 runs

Spatial Bayesian inferences in GENELAND resulted in 41 of 50 runs (82%) of modal value at four subpopulations, whereas seven runs gave a mode at three for the posterior distribution of K. Obtained results supported the distinction of samples collected in the northern part of study area. Individuals from Pisz, Borki, Rominta and northern part of Augustów Forests were consistently clustered together (G1 in Figure 5). The other subpopulations were as follow: G2—southern part of Augustów Forest and Augustów–Knyszyn Transect, G3—Knyszyn and Białowieża Forests, Knyszyn–Białowieża and Białowieża–Mielnik Transects, and G4—Mielnik Forest (Figure 5). Analysis in TESS (data not shown) defined four spatial groups in agreement with the clusters identified by GENELAND in 41 runs. Genetic differentiation (*F*_ST_) between the clusters (G1–G4) showed low values from 0.018 to 0.032, with the highest one between the most geographically distant subpopulations G1 and G4 (Supporting Information Table S5).

### 3.2 Genetic similarity among mice in relation to geographic distance and population abundance

The population abundance index of yellow‐necked mice varied from 1.94 to 9.37 per 100 trapnights (Supporting Information Table S1). Such a wide range suggests huge differences in population density. Jędrzejewski, Jędrzejewska, and Szymura (1995) have shown that linear increase in abundance index between two and nine yellow‐necked mice per 100 trapnights reflects the exponential increase in absolute population density between 5–10 and 100–200 mice per hectare. Efective population size (*N*_e_) ranged from 33.2 to 281.6 (Supporting Information Table S1) and was positively correlated to the index of mice abundance (*r* = 0.70, *p* = 0.02). In our data, population abundance of mice was positively related to the share of deciduous forest in each region (*Y* = 2.23ln(*x*) − 0.23, *R* ^2^ = 0.64, *p* = 0.005, Supporting Information Figure S1 upper panel), where *Y*—number of mice per 100 trapnights, *x*—percent area covered by deciduous forests. The same trend, although not significant statistically, was found between effective population size and the share of deciduous forest in each region (*R* ^2^ = 0.37, *p* = 0.06, Supporting Information Figure S1 lower panel).

Genetic differentiation (*F*_ST_) between mouse populations in 10 regions was highly correlated with geographic distance (*r* = 0.78, *p* = 0.001). This correlation, calculated at the individual level, although still significant, was substantially weaker (*r* = 0.112, *p* = 0.001) and exhibited linear relationship only at very short geographic distances.

Spatial autocorrelation analysis, which evaluates the correlation between genetic similarity and geographic distance, yielded significant positive autocorrelation *r*‐values over relatively long distances (Supporting Information Figure S2). Despite high variation among regions, the first distance class (1 km) had significant autocorrelation r‐values in all studied forests and transects. Individuals from Pisz Forest showed significant autocorrelation within 1‐km distance class only, whereas mice from Białowieża Forest exhibited significant *r*‐values up to 12‐km distance classes (Supporting Information Figure S2). The maximum distance, at which significant autocorrelation was observed, was 14 km for individuals from Augustów–Knyszyn Transect. There was no clear spatial pattern found with high variation observed among geographical regions (Supporting Information Figure S2).

**Figure 6:**
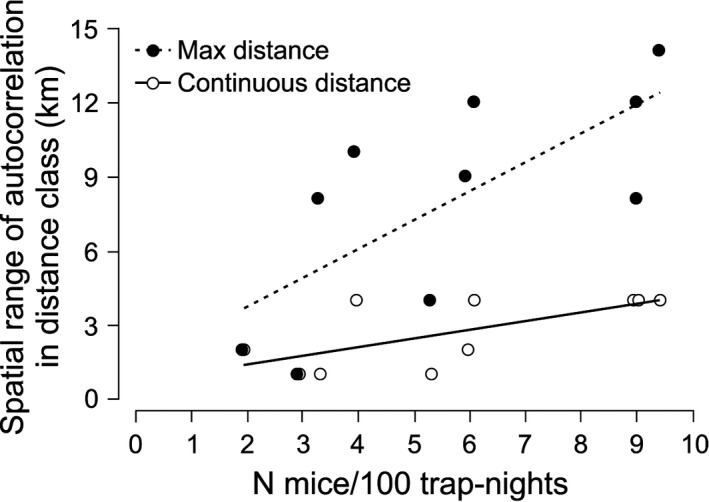
Dependence of the spatial range of autocorrelation r‐values on estimated abundance of yellow‐necked mouse in the studied regions; Max distance—maximum distance class (in km), at which significant positive autocorrelation was found. Continuous distance—number of 1‐km distance classes with continuous positive autocorrelation (with only one insignificant class, separating significant *r*‐values, acceptable). Max distance: *Y* = 1.164*x* + 1.408, *R* ^2^ = 0.51, *p* = 0.02; Continuous distance: *Y* = 0.355*x* + 0.688, *R* ^2^ = 0.46, *p* = 0.03. Sources of data in Supporting Information Figure S2 and Table S1

Therefore, in a further step two measures were included as follows: (a) the number of 1‐km classes with continuous positive autocorrelation found (with only one insignificant class, separating significant *r*‐values, acceptable), and (b) the maximum distance class, at which positive autocorrelation was found. These two measures were compared with the mean abundance indices of mice in 10 regions. The observed pattern of fine‐scale spatial structure was significantly related to mouse abundance (continuous distance: *R* ^2^ = 0.46, *p* = 0.03, max distance: *R* ^2^ = 0.51, *p* = 0.02; Figure 6). The higher was the abundance of mice, the larger was the spatial scale of their autocorrelation.

For a precise assessment of the gradual change in genetic similarity/dissimilarity values, two pairwise statistics (Queller and Goodnight relatedness coefficient, Rousset's genetic distance a_r_) were calculated between all trapped individuals within the first 6 km for mice in woodlands and 25 km for mice in transects. In woodlands, the greatest change of values occurred between the first and the second distance class (0–1 km) (Supporting Information Figures S3 and S4). However, these changes were not identical in all regions. Mice from Białowieża and Mielnik Forests exhibited constant level of relatedness (and differentiation) in all six classes of distances, whereas individuals from Rominta, Borki, and Augustów Forests showed more rapid changes of these genetic parameters. There was also a substancial difference between forests in the first distance class: pairwise relatedness coefficient ranged from 0.027 to 0.173 with the least related individuals found in Białowieża and Mielnik Forest and the most related in Augustów Forest (Supporting Information Figure S4). Accordingly, the lowest genetic distance occurred between mice in Augustów Forest (*a*_r_ = −0.064) and the highest in Białowieża and Mielnik Forests (*a*_r_ = 0.078 and 0.089, respectively) (Supporting Information Figure S3). Mean relatedness coefficients and genetic distances calculated for mice captured along transects had moderate values, within the range found in forests. Relatedness level in the first distance class ranged from 0.034 to 0.093 and genetic distance from 0.029 to 0.052 (Supporting Information Figure S5).

**Figure 7:**
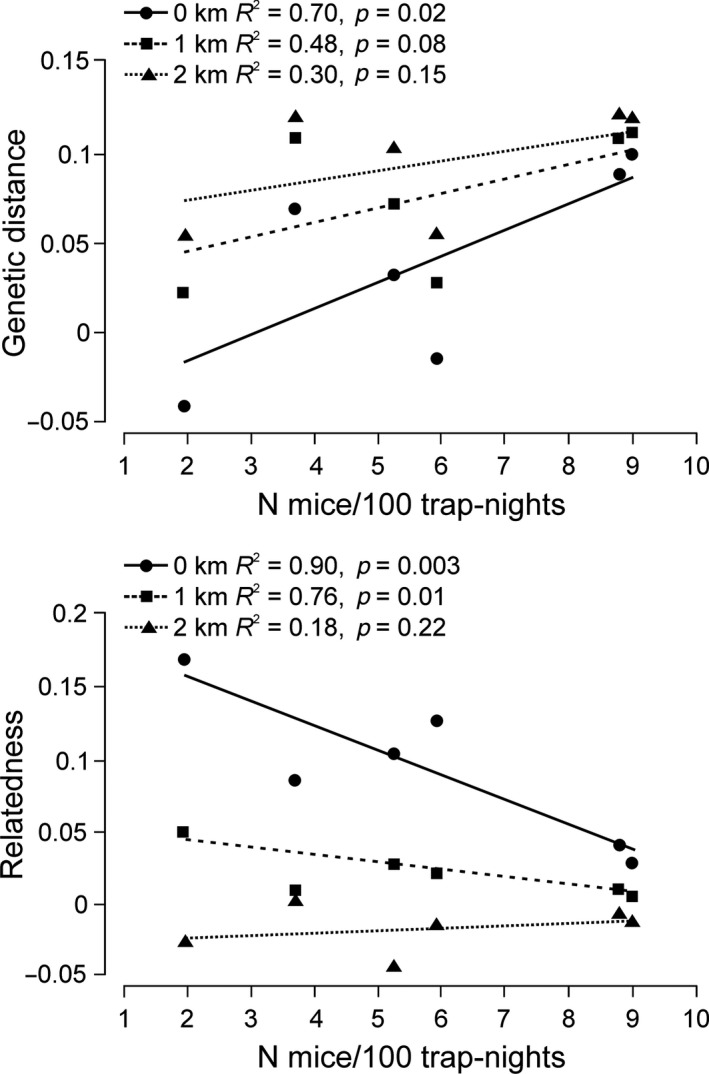
Mean values of Rousset's genetic distances (a_r_—upper panel) and Queller and Goodnight relatedness coefficients (lower panel) calculated for individuals from six forests (PIS excluded due to small sample size) in three distance classes in relation to abundance of yellow‐necked mice. Sources of data in Supporting Information Figures S3 and S4 and Table S1

In the six forests (PIS excluded due to small sample size), individual genetic distances were significantly positively associated with an abundance index of mice at the first distance class (*R* ^2^ = 0.70, *p* = 0.02), the relationship got weaker at 1‐km distance, and disappeared at 2 km (Figure 7). Dependence of pairwise relatedness on abundance of mice was even higher, with significant relationship at the first and second distance classes (Figure 7). Interestingly, high level of genetic similarity was negatively associated with increasing abundance of mice. The relationship between abundance of mice and their pairwise relatedness/genetic distance was not visible for data collected along three transects (Supporting Information Figure S5).

**Figure 8:**
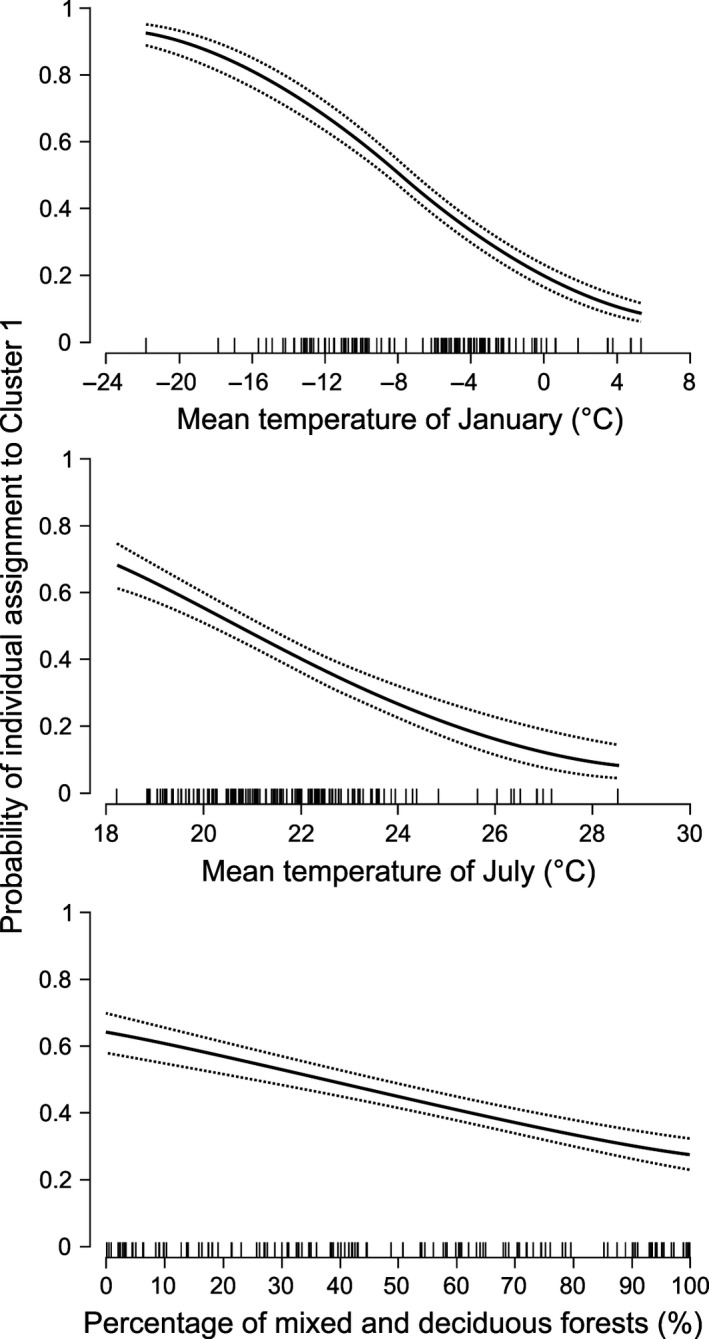
Relationships between mean individual assignment of individual yellow‐necked mice to Cluster 1 (based on Structure results) and mean temperature of January, mean temperature of July, and percentage of mixed and deciduous forests in the trapping sites—results of the most parsimonous model presented in Table 4

In our testing the association between environmental variables and genetic assignment of yellow‐necked mice to Cluster 1, the AIC ranking indicated that—among all the combinations of considered submodels—the model with all explanatory variables had the lowest AIC_c_ scores. Therefore, we selected this submodel as a single best model (Table 3). The GLMM showed a negative effect of growing temperatures (both January and July) on the probability of genetic assignment of mice to Cluster 1 (*p *<* *0.001; Table 4, Figure 8). In addition, the prevalence of Cluster 1 decreased with the growing share of deciduous‐mixed forests (*p *<* *0.001). Winter severity (approximated by mean temperature of January) had the strongest impact on Cluster 1 spatial distribution (see *z*‐values in Table 4). In general, mice belonging to Cluster 1 prevailed in colder regions and in the boreal‐type, coniferous forests.

**Table 3:** Model selection (based on the AIC_c_ criteria) for the considered GLMMs. The models aimed at assessing the effects of mean temperature of January (*T*_JAN_), mean temperature of July (*T*_JUL_), and the percentage of deciduous‐mixed forests (DMF) on the probability of genetic assignment of individual mice to Cluster 1 (see Figure 3; green color)

Model	*K*	AIC_c_	ΔAIC_c_
*T*_JAN_ + *T*_JUL_ + DMF	5	9,940.2	0
*T*_JAN_ + DMF	4	9,983.3	43.12
*T*_JAN_ + *T*_JUL_	4	9,989.8	49.58
*T*_JAN_	3	10,016.1	75.91
*T*_JUL_ + DMF	4	10,080.6	140.39
*T*_JUL_	3	10,104.5	164.26
DMF	3	10,105.0	164.83
Intercept	2	10,120.6	180.37

*K*: the number of estimated parameters; AIC_c_: Akaike's Information Criterion with a second‐order correction for small sample sizes; ΔAIC_c_: difference in AIC_c_ between the specific model and the most parsimonious model.

**Table 4:** Parameter estimates for the generalized linear mixed‐effects model (GLMM; indicated as most parsimonious by AIC) describing the effects of mean temperature of January, mean temperature of July and the percentage of deciduous‐mixed forests, on the probability of genetic assignment of individual mice to Cluster 1

Variables	Estimate	*SE*	*z* Value	*p* Value
Intercept	6.11	1.00	6.10	<0.001
Mean temperature of January	−0.18	0.01	−12.52	<0.001
Mean temperature of July	−0.31	0.04	−6.82	<0.001
Percentage of deciduous‐mixed forests	−1.59	0.22	−7.31	<0.001

## 4 DISCUSSION

### 4.1 Genetic variation and differentiation among local populations

Genetic variation of yellow‐necked mice in NE Poland, estimated using 13 microsatellite markers, was very high in all studied regions. All markers were highly polymorphic and allelic richness indicated similarly high level of genetic variation. Such maintenance of high genetic variation, despite great fluctuations in population density and thus possible threat of repetitive bottlenecks, has already been observed in yellow‐necked mouse (Gortat et al., 2010; Kozakiewicz et al., 2009; Rico et al., 2009) and in several vole species (e.g., Aars et al., 2006; Berthier et al., 2005, 2006; Ehrich & Jorde, 2005; Ehrich et al., 2009; Gauffre, Estoup, Bretagnolle, & Cosson, 2008; Gauffre et al., 2014; Plante, Boag, & Bradley, 1989; Redeker et al., 2006; Rikalainen et al., 2012; Vuorinen & Eskelinen, 2005). The authors explained a minor impact of often dramatic decline in population size (up to 90% of population; Rikalainen et al., 2012) by constant and relatively large effective population size, intense migration negatively correlated with density, and the consequential accumulation of new alleles during the population peaks.

Interestingly, mice inhabiting Rominta and Białowieża Forests exhibited the highest number of private alleles. Detection of such alleles in Białowieża Forest might indicate that there is a slight isolation and a limited exchange of individuals between Białowieża subpopulation and adjacent areas. It was also confirmed by relatively high F_IS_ value observed there. If dispersal is highly sex‐biased (mainly male‐biased in mammals, Greenwood, 1980) one might expect negative *F*_IS_ values as a result of mating between individuals originating from different source populations (Prout, 1981). Such phenomenon is a common feature in fragmented vole populations (e.g., Ratkiewicz & Borkowska, 2006) where high dispersal rate is negatively correlated with population size. Lack of negative values in our data might indicate that there is no sex bias in dispersal rate in the yellow‐necked mouse, low number of immigrants are able to reproduce in favorable habitat (e.g., Białowieża Forest; *F*_IS_ = 0.101) or individuals from adjacent areas that successfully reproduce are genetically similar. It was found in voles trapped in Białowieża meadow that recruitment of individuals from outside did not substantially affect the genetic results as they exhibited highly similar genetic profiles (Pilot et al., 2010).

Deviation from Hardy–Weinberg equilibrium observed in our data is reasonable and results from the assumption of the theoretical model that is rarely met in natural populations. The geographical scale of the study area greatly exceeded the potential for unlimited dispersal of individuals. However, when HWE was tested in a smaller scale, substantially lower number of loci exhibited significant excess of homozygotes. Similar results were found in the yellow‐necked mouse population investigated by Kozakiewicz et al. (2009) and Gortat et al. (2010). In their analyses, all samples from Pisz Forest pooled together resulted in two loci deviating significantly from HWE, whereas such deviation was not found when analyses were conducted at the very fine scale.

Genetic differentiation among the studied regions was low but significant. Hierarchical population structure was found. Primal genetic subdivision was observed between the northern and the southern parts of the study area. The obtained results on population genetic structure were highly concordant among applied nonspatial (STRUCTURE, PCA) and spatial (GENELAND, TESS) methods. Clear genetic distinction of northern subpopulation (Cluster 1, G1) was supported by all programs. The remaining samples with more continuous spatial distribution were less genetically structured, and formed a gradient but with several detected areas of disruption in gene flow, which resulted in a more subtle but still visible differentiation.

Low values of *F*_ST_ (between geographical regions and genetic subpopulations) observed in this study are in a range of values found in similarly abundant and highly variable small mammal species. Nagylaki (1998) and Hedrick (1999) argued that *F*_ST_ may be a poor measure of genetic differentiation, when the level of diversity in the markers considered is very high. Jakobsson, Edge, and Rosenberg (2013) have further demonstrated how the variation in profiles of the rare and the most frequent alleles, within and among compared populations, can constrain the range of F statistics. He concluded that many unusual observations of *F*_ST_ can be understood not as biological phenomena but rather as consequences of the mathematical dependence of *F*_ST_ on the properties of allele‐frequency distributions. In a large‐scale study on voles in France, Gauffre et al. (2008) found that *F*_ST_ calculated between populations across the motorway was similarly high to genetic differentiation detected between distant local populations not separated by any kind of barrier. He suggested careful interpretation of genetic distinction based solely on *F*_ST_ as isolation by distance might produce values that confound the results.

### 4.2 Factors explaining genetic variation pattern

Identification of ecological variables that contribute to population genetic divergence is a primal goal in landscape genetics (Manel, Schwartz, Luikart, & Taberlet, 2003). Although the impacts of various features are not mutually exclusive and spatial genetic pattern usually results from combination of several factors, we made the attempt to evaluate the role of each category of factors separately.

At the larger scale, geographic distance was found to be the main factor shaping a nuclear genetic variation. It was supported by *F*_ST_ values, PCA plot structure and the gradient of genetic differentiation. Nevertheless, such relationship between genetic and geographic distance was much weaker, and held the linearity at very fine scale (~1.5 km), when analyses were conducted at individual level. Such incoherent results are in high concordance with published data on small mammals (e.g., Trizio et al., 2005). Despite unrealistic assumptions of the theoretical model introduced by Wright (1943), isolation by distance (IBD) is still commonly used by population genetists to describe spatial pattern of genetic variation and such correlation is indeed often found (Jenkins et al., 2010 and reference therein). However, genetic IBD patterns may not necessarily be observed even if dispersal is actually restricted in space (Guillot, Leblois, Coulon, & Frantz, 2009; Leblois et al., 2004; Rousset, 1997). Lack of genetic IBD pattern is usually explained as results of substantial, long‐distance dispersal (Ehrich et al., 2001; Redeker et al., 2006; Schweizer et al., 2007), dispersal barriers (Ratkiewicz & Borkowska, 2006), large effective population size (Guivier et al., 2011) or migration‐drift disequilibrium (Ehrich & Stenseth, 2001; Francl, Glenn, Castleberry, & Ford, 2008).

Overall high genetic variation of mice in NE Poland and their consistent genetic structure across consecutive years indicated that fluctuations in population size had no serious impact on genetic parameters. Most probably it is due to a large effective population size, which can buffer population response to evolutionary forces. The differences in habitat composition (the share of deciduous forest) among the sampled areas had a profound impact on population density indices, what subsequently influenced kinship and spatial autocorrelation results. The decrease of relatedness in dense population was observed also in the root vole *Microtus oeconomus* (Pilot et al., 2010) and it was suggested that with increasing density more kin groups occupy the same space. On a contrary, in years with sparse distribution of individuals, one family group can span over larger area what results in higher estimates of mean relatedness. Positive autocorrelation was also found to be highly associated with population density, but it is unclear whether the stronger population structure resulted from dispersal rate or favorable habitat share.

Winter severity was found to be the main factor that explained the spatial distribution of the two main genetic clusters of mice in NE Poland. Although the pattern revealed by microsatellites is believed to reflect the neutral genetic variability, it can be highly consistent with the adaptive variability as has been shown for European wolves *Canis lupus* (Stronen et al., 2013, 2015). It was already found by Wójcik (1993) that—in a population inhabiting Białowieża Forest—mice surviving winter were not a random representation of population in respect of polymorphism in the transferrin locus. Heterozygotes had a selective advantage in winter survival over homozygotes.

## 5 CONCLUSIONS

In NE Poland, the yellow‐necked mouse population exhibited a major division into two genetic clusters. Their spatial distribution was correlated to environmental factors, mainly winter severity. Fine‐scale genetic structure was highly influenced by mouse population abundance, which was determined by the share of optimal habitats (deciduous forests).

## CONFLICT OF INTEREST

None declared.

## AUTHORS’ CONTRIBUTIONS

BJ, MN, and SDC conceived the ideas and applied for the financial support, SDC and MN collected the samples, SDC, BJ, and TB analyzed the data, and SDC, MN, and BJ wrote the paper.

## DATA ACCESSIBILITY

Sampling locations, ecological and climatic data and microsatellite genotypes supporting the results are deposited in Dryad repository: https://doi.org/10.5061/dryad.f8q8qf5.
