# Supplementary material for: Regional and local patterns of genetic variation and structure in yellow‐necked mice ‐ the roles of geographic distance, population abundance, and winter severity
Source: Ecol Evol. 2018 Jul 22;8(16):8171–86. doi: 10.1002/ece3.4291 (PMC6145024; doi:10.1002/ece3.4291)
Supplement: Supplementary file 1 [file ECE3-8-8171-s001.docx]

**Supplementary material**

**Regional and local patterns of genetic variation and structure in yellow-necked mice − the roles of geographic distance, population abundance and winter severity**

Sylwia D. Czarnomska, Magdalena Niedziałkowska, Tomasz Borowik, Bogumiła Jędrzejewska

Table S1. Ecological characteristics of the studied forests and transects and abundance indices of yellow-necked mice. See Fig 2 for abbreviations of regions and their location. Percentage of land use categories based on Corine Landcover 2006 (CLC2006) data in 1-km buffer zone around each trapping site. Mean temperature collected based on four measurements per month of land surface temperature in a spatial grid 1 km × 1 km (MODIS). Mean abundance index calculated as a number of animals captured per 100 trapnights. Effective population size (Ne) calculated using a bias corrected version of the linkage disequilibrium method of Waples and Do 2008 with the option of random mating selected using NeEstimator V2.1 with frequency critical value of 0.05 (Do et al. 2014).

| Region | Mean abundance index  N mice/100 TN | | N_e_ | Mean temperature (°C) | |  | | Percentage cover of land use category | | | | | | |  |
| --- | --- | --- | --- | --- | --- | --- | --- | --- | --- | --- | --- | --- | --- | --- | --- |
|  |  |  |  | January | July |  | Coniferous  forest | | Mixed forest | Deciduous  forest | Meadows and arable land | Waters | | Other | |
| AUG | 1.94 | 35.5 | | –5.26 | 22.52 |  | 50.5 | | 31.3 | 4.0 | 7.5 | 3.5 | | 3.2 | |
| BIAL | 8.93 | 257.9 | | –2.74 | 20.71 |  | 29.6 | | 27.9 | 41.3 | 1.2 | 0 | | 0 | |
| BOR | 5.27 | 72.3 | | –6.80 | 21.87 |  | 15.1 | | 63.4 | 12.4 | 0.6 | 6.8 | | 1.7 | |
| KNYSZ | 6.04 | 151.1 | | –4.71 | 22.38 |  | 58.4 | | 16.9 | 8.3 | 12.2 | 0 | | 4.2 | |
| MIEL | 8.99 | 281.6 | | –9.85 | 19.93 |  | 24.3 | | 5.6 | 65.4 | 2.8 | 0 | | 1.9 | |
| ROM | 5.93 | 50.4 | | –11.40 | 20.58 |  | 16.4 | | 48.0 | 25.5 | 7.4 | 0 | | 2.7 | |
| PIS | 2.92 | 33.2 | | –6.79 | 21.33 |  | 46.0 | | 26.5 | 15.8 | 7.9 | 1.7 | | 2.1 | |
| Mean for Forests | – | - | | –6.79 | 21.33 |  | 34.3 | | 31.4 | 24.7 | 5.6 | 1.7 | | 2.3 | |
| TAK | 9.37 | 108.5 | | –6.41 | 22.79 |  | 18.4 | | 11.1 | 25.4 | 42.5 | 0 | | 2.6 | |
| TKB | 3.29 | 144.5 | | –8.43 | 22.35 |  | 59.4 | | 2.3 | 8.0 | 26,2 | 1.4 | | 2.7 | |
| TBM | 3.93 | 88.6 | | –7.24 | 22.24 |  | 53.4 | | 5.6 | 3.3 | 25.8 | 0 | | 11.9 | |
| Mean for Transects | – | – | | –7.36 | 22.46 |  | 43.7 | | 6.3 | 12.2 | 31.5 | 0.5 | 5.7 | | |

Table S2. Number of samples (individuals of yellow-necked mice) successfully analysed per geographical region.

| Region | Number of samples |
| --- | --- |
| Augustów Forest  2006 | 7 |
| 2007 | 17 |
| 2008 | 17 |
| Białowieża Forest  2004 | 2 |
| 2005 | 9 |
| 2007 | 76 |
| 2008 | 67 |
| Borki Forest  2004 | 50 |
| 2006 | 3 |
| Knyszyn Forest  2004 | 2 |
| 2006 | 14 |
| 2007 | 34 |
| 2008 | 30 |
| Mielnik Forest  2005 | 20 |
| 2006 | 20 |
| 2007 | 45 |
| 2008 | 74 |
| Pisz Forest 2004 | 15 |
| Rominta Forest  2004 | 37 |
| 2006 | 17 |
| Augustów – Knyszyn Transect  2007 | 38 |
| 2008 | 63 |
| Knyszyn – Białowieża Transect 2007 | 52 |
| 2008 | 3 |
| Białowieża – Mielnik Transect 2007 | 42 |
| 2008 | 14 |
| Total | 768 |

Table S3. Detailed information on genetic variability of the analysed loci in the yellow-necked mouse population in NE Poland.

| Marker ID | Number of alleles  (N_a_) | Null allele frequency | Observed heterozygosity (H_o_) | Expected heterozygosity (H_e_) |
| --- | --- | --- | --- | --- |
| AF246520 | 20 | 0.036 | 0.833 | 0.902 |
| AF246522 | 26 | 0.102 | 0.717 | 0.913 |
| AF246523 | 16 | 0.015 | 0.832 | 0.859 |
| MSAF–7 | 17 | 0.078 | 0.749 | 0.897 |
| MSAF–3 | 21 | 0.138 | 0.663 | 0.929 |
| MSAF–16 | 25 | 0.008 | 0.878 | 0.892 |
| MSAF–8 | 31 | 0.029 | 0.878 | 0.934 |
| MSAF–22 | 32 | 0.041 | 0.853 | 0.934 |
| GTTC4A | 19 | 0.048 | 0.710 | 0.796 |
| GACAD1A | 26 | 0.062 | 0.741 | 0.856 |
| CAA2A | 18 | 0.031 | 0.822 | 0.880 |
| GCATD7S | 28 | 0.049 | 0.765 | 0.921 |
| TNF | 15 | –0.014 | 0.856 | 0.894 |

Table S4. Results of calculations for the number of genetic clusters indicated by STRUCTURE using the method of Evanno *et al*. (2005). K – number of clusters, Ln P(D) – mean value of posterior probability of K calculated in STRUCTURE for different K values (1–12), ΔK second order of change of the log probability of data between successive K values. In bold the most probable number of subpopulations and the highest values of Ln P(D) and ΔK*.*

| K | Ln P(D) | ΔK |
| --- | --- | --- |
| 1 | –49307.29 | – |
| 2 | –48633.42 | **176.12** |
| 3 | –48269.24 | 7.10 |
| 4 | **–47930.82** | **31.59** |
| 5 | **–47747.65** | **55.25** |
| 6 | –48137.19 | 0.71 |
| 7 | –48127.28 | 0.16 |
| 8 | –48359.44 | 0.47 |
| 9 | –49256.62 | 0.74 |
| 10 | –48948.04 | – |

Table S5. Pairwise genetic differentation F_ST_ among four subpopulations (G1–G4, see Fig. 5) defined using spatial Bayesian methods (GENELAND, TESS). All values are statistically significant at p < 0.008 after Bonferroni correction (k=6).

| Subpopulation | G1 | G2 | G3 |
| --- | --- | --- | --- |
| G2 | 0.022 | – |  |
| G3 | 0.030 | 0.020 | – |
| G4 | 0.032 | 0.028 | 0.018 |


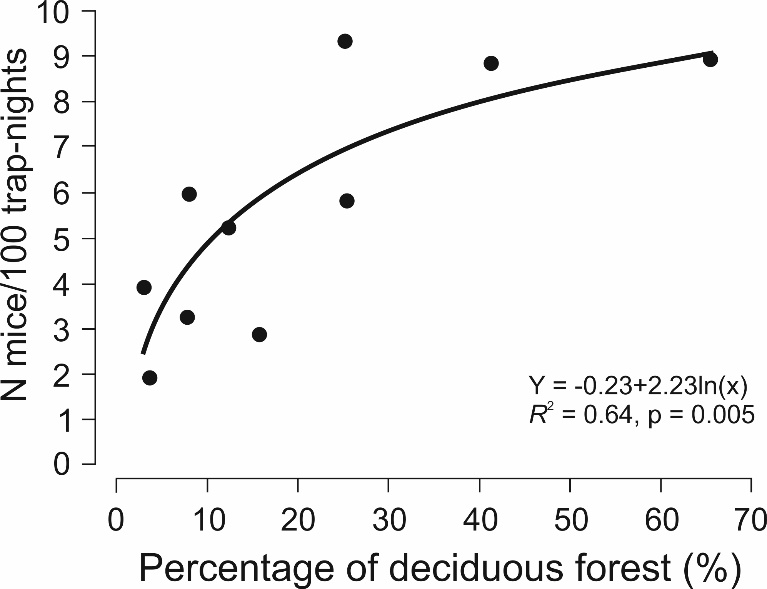


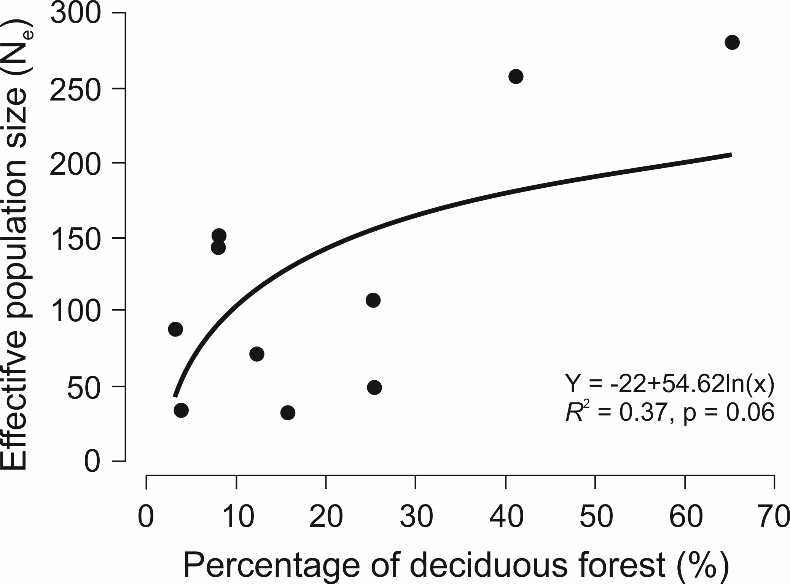


Fig S1. Relationship between mean abundance index of mice (upper panel), effective population size (lower panel) and the share of deciduous forest in each of 10 geographical regions (data in Table S1).

Pisz Forest


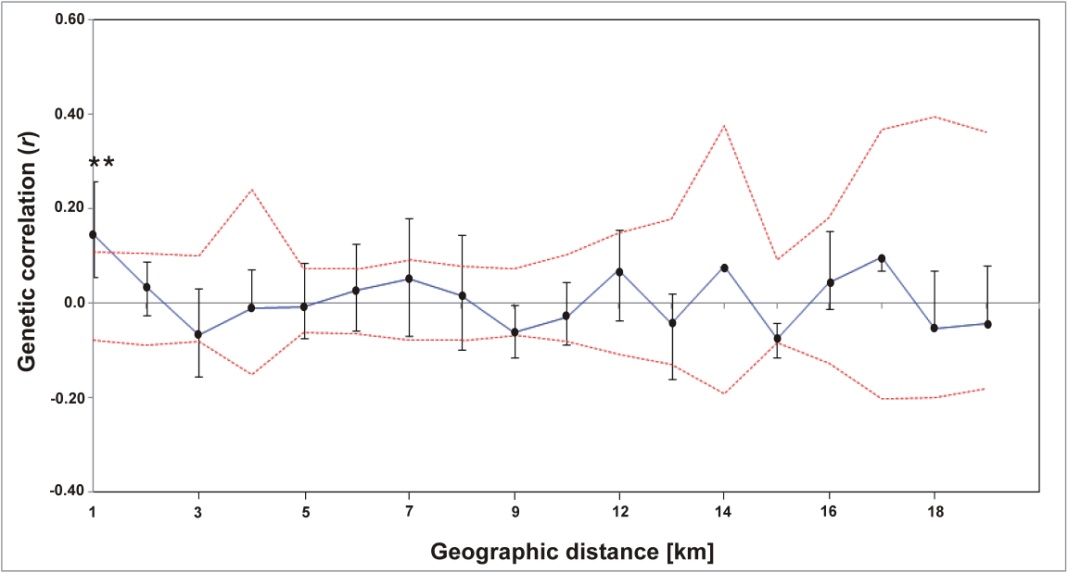


Borki Forest


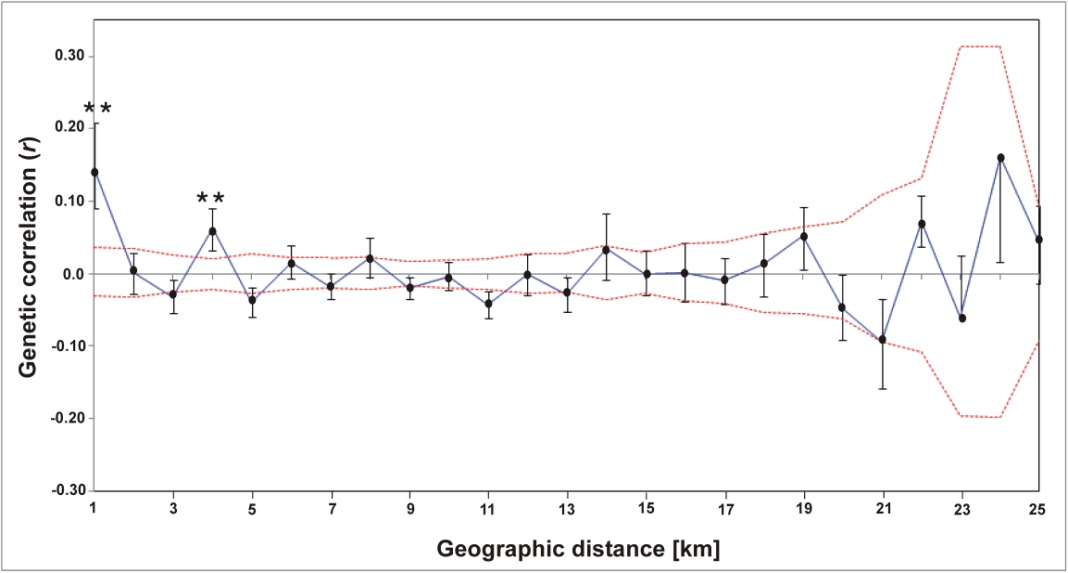


Rominta Forest


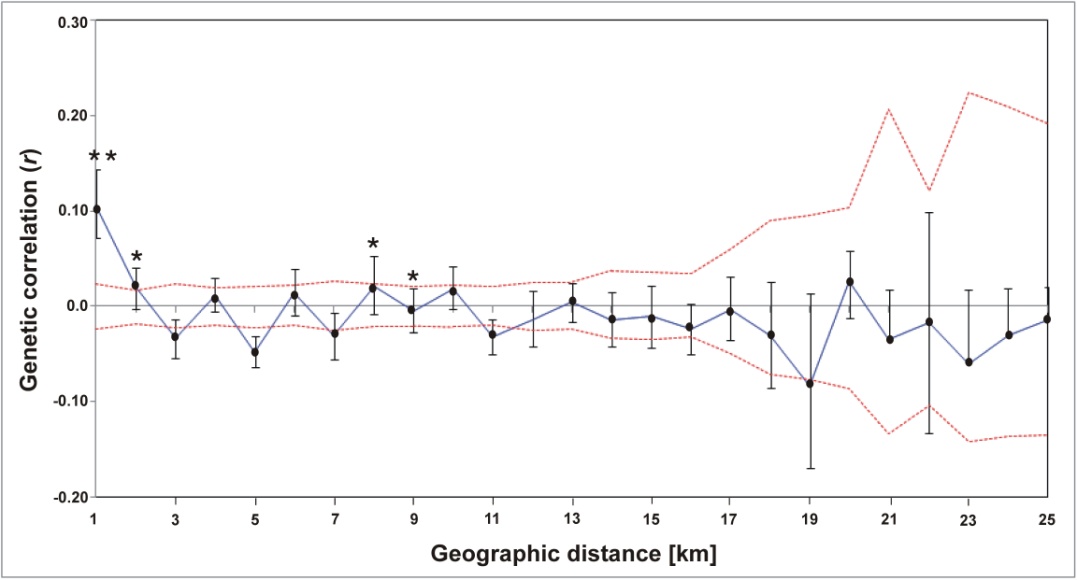


Augustów Forest

**
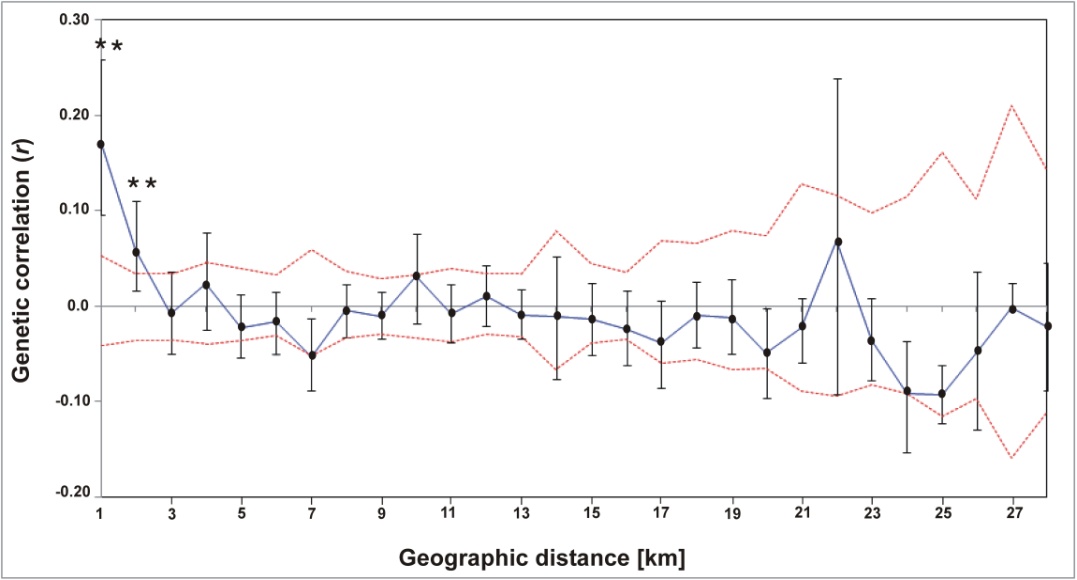
**

Knyszyn Forest


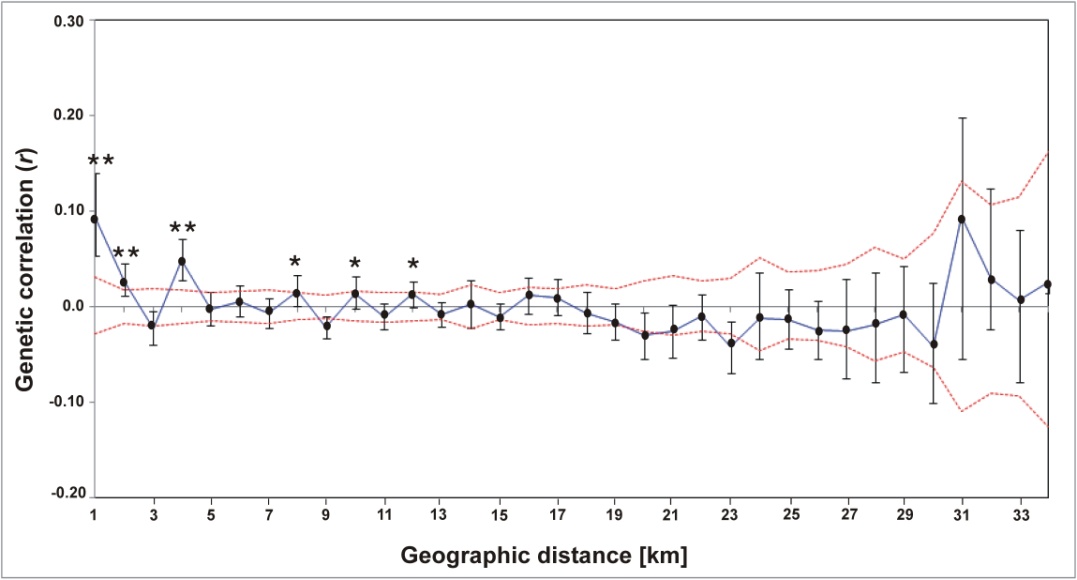


Białowieża Forest


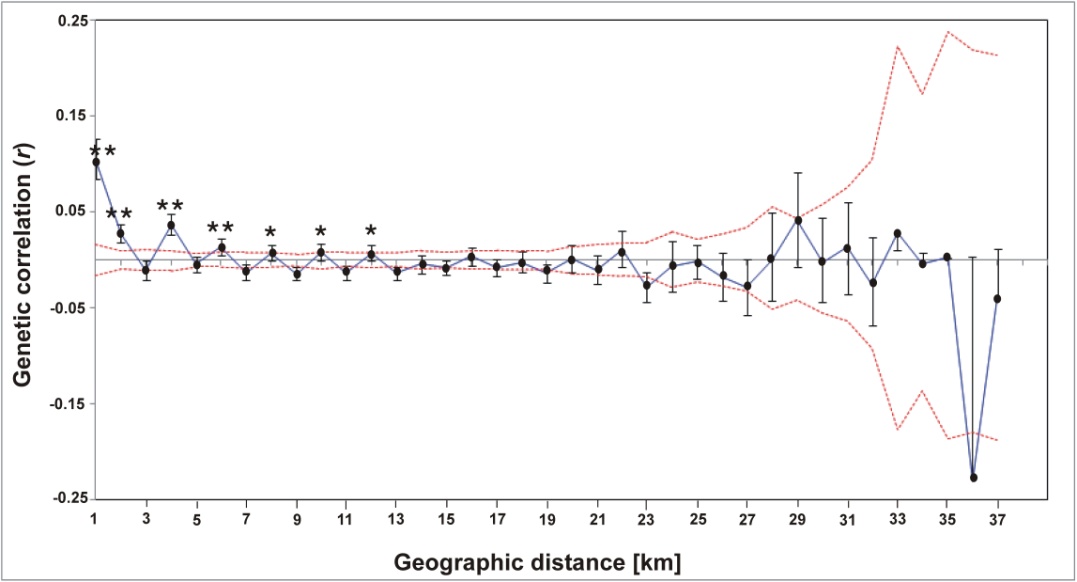


Mielnik Forest


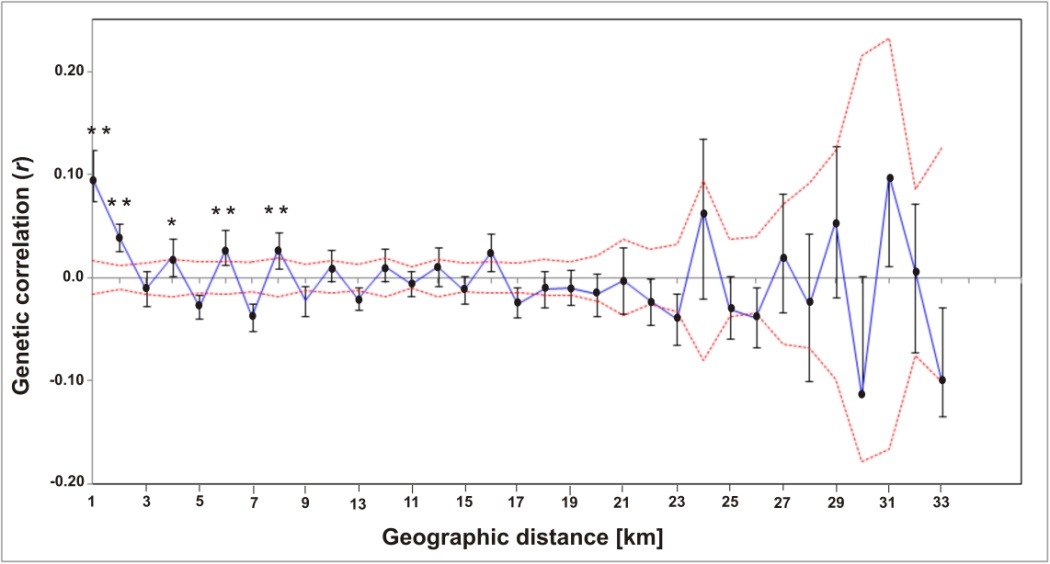


Augustów Forest – Knyszyn Forest Transect


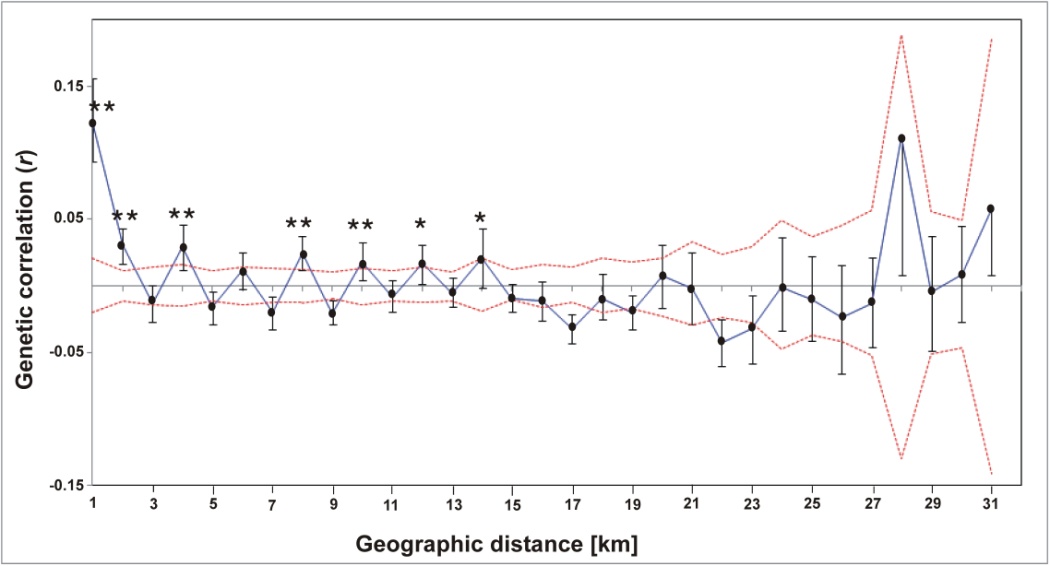


Knyszyn Forest – Białowieża Forest Transect


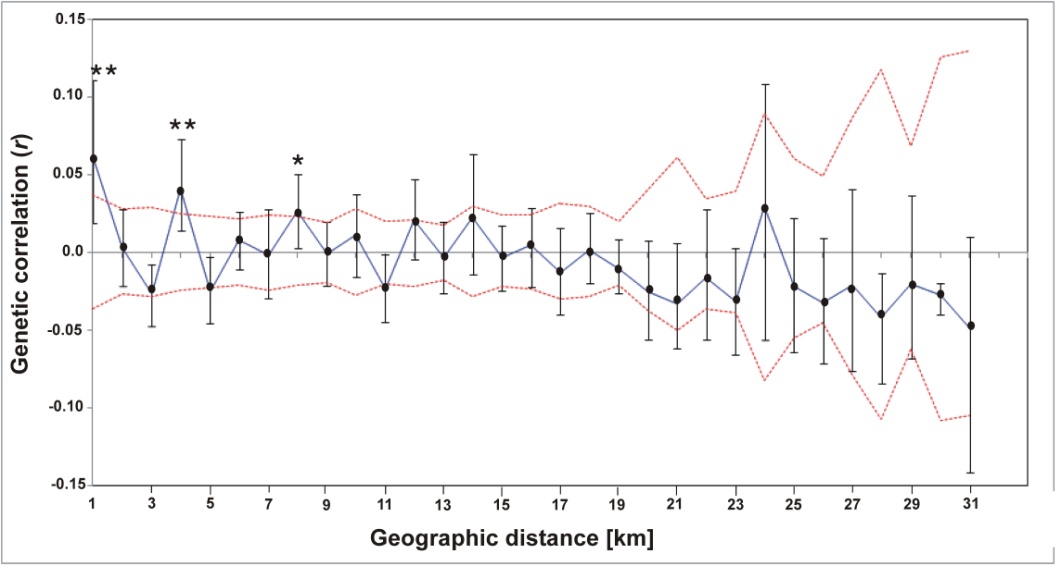


Białowieża Forest – Mielnik Forest Transect


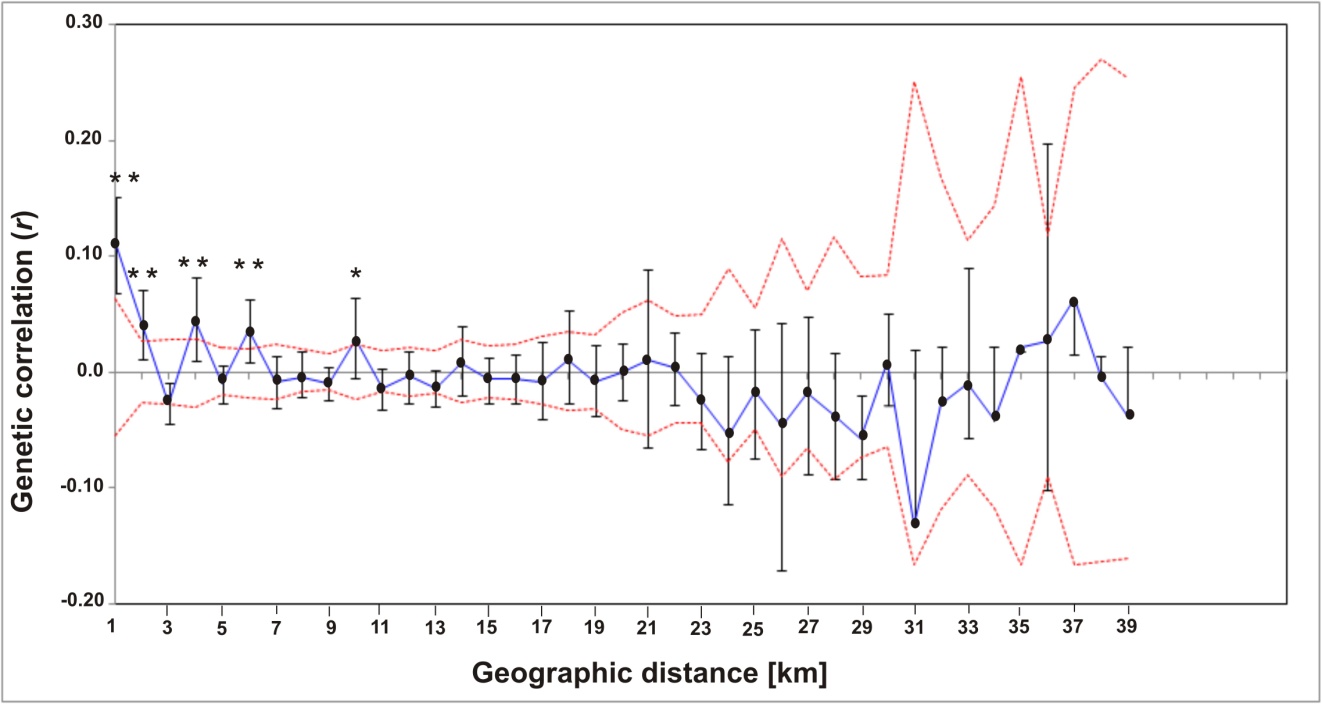


Fig. S2. Spatial autocorrelation results of the genetic structure of the yellow-necked mouse population calculated for each geographical region. Numbers of distance classes differ among regions due to the difference in spatial scale covered by sampling. Each class equals 1 km. Stars indicated statistically significant positive spatial autocorrelation values (*p < 0.05; **p < 0.01). The permutation 95% confidence interval (dashed lines) and the bootstrapped 95% confidence error bars are also shown.


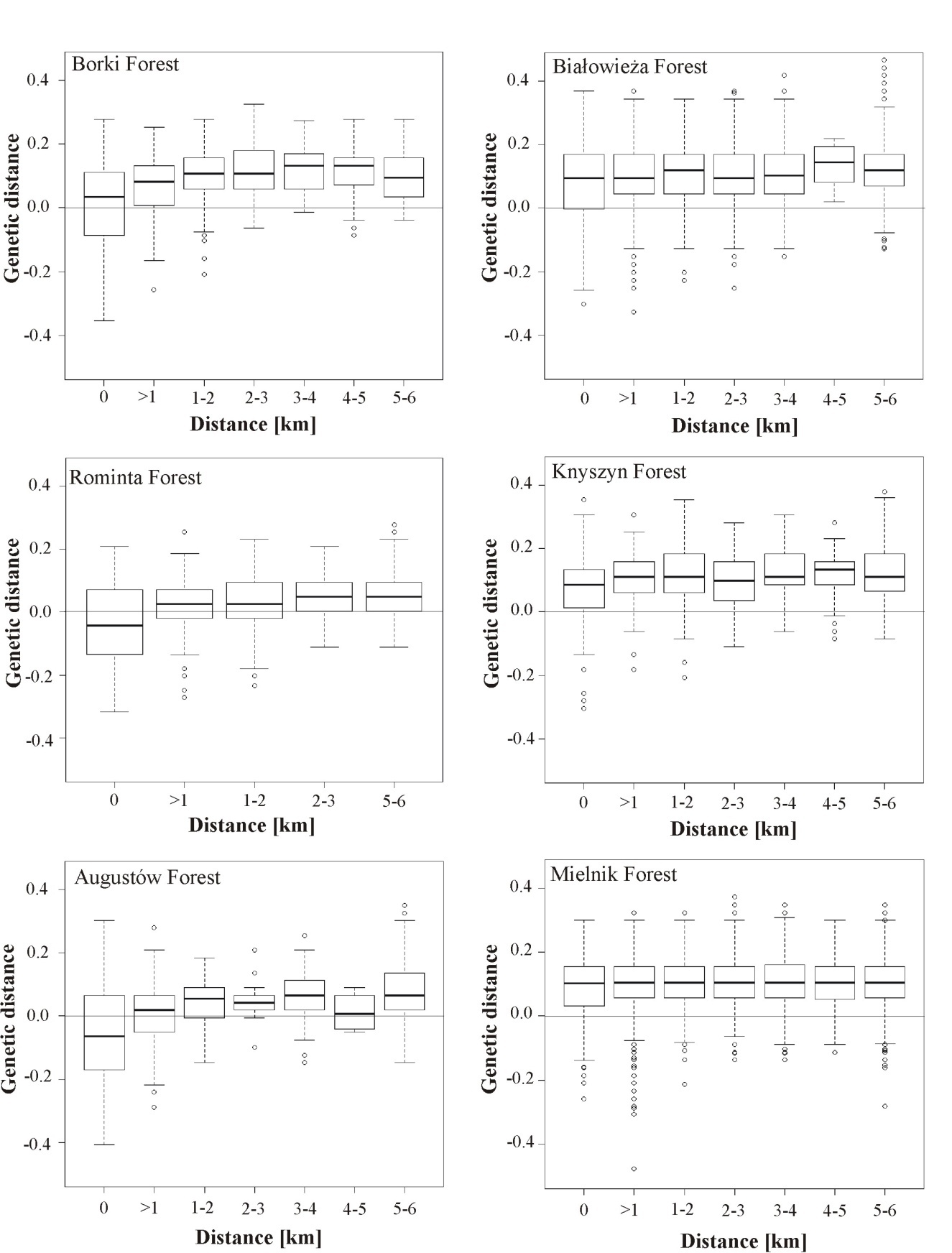


Fig. S3. Rousset’s individual genetic distance (a_r_) calculated in six distance classes
for yellow-necked mice collected in six forests. Samples from Pisz Forest excluded due to small sample size (n = 15).


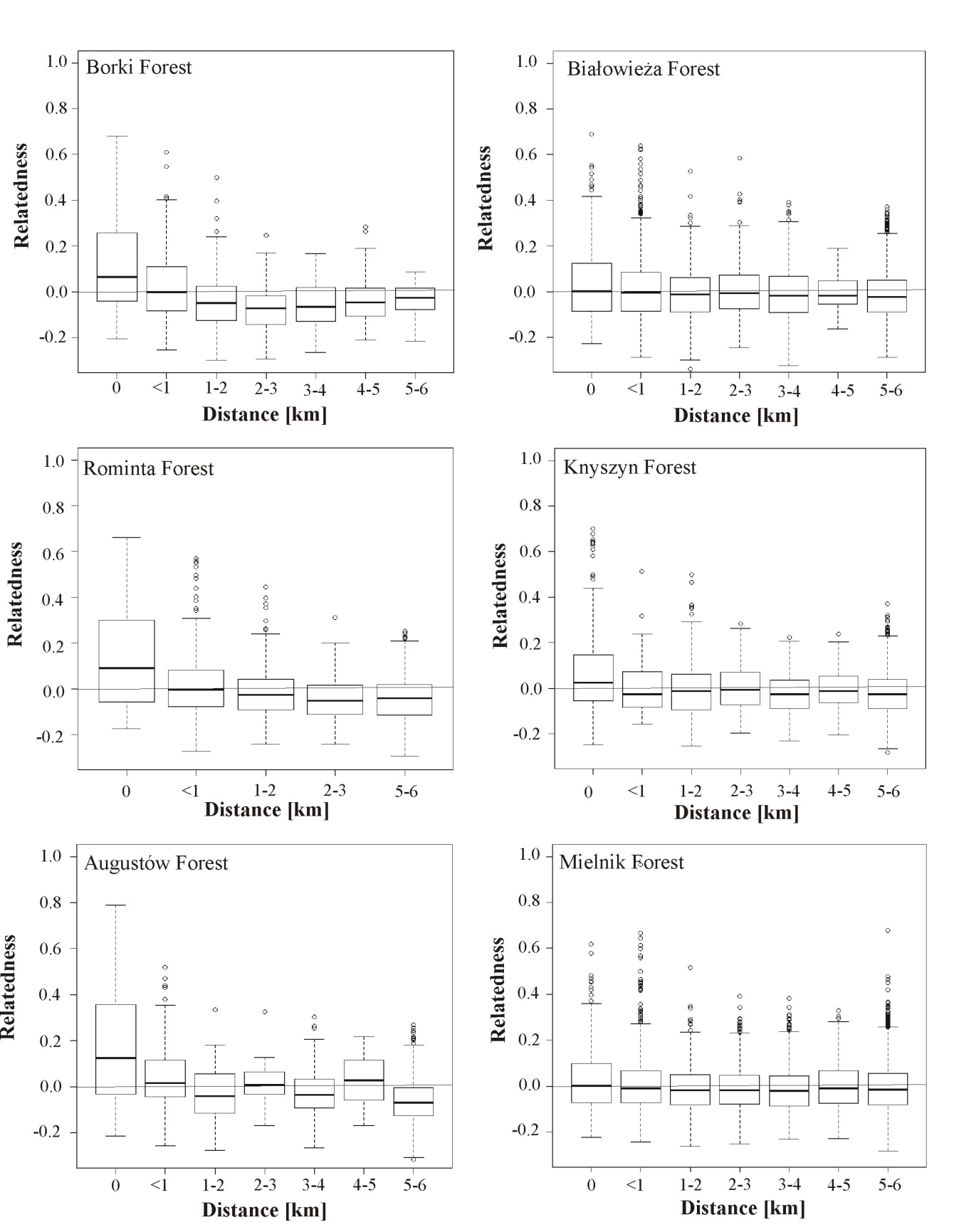


Fig. S4. Queller and Goodnight relatedness coefficient calculated in six distance classes
for yellow-necked mice collected in six forests. Pisz Forest excluded due to small sample size (n = 15).


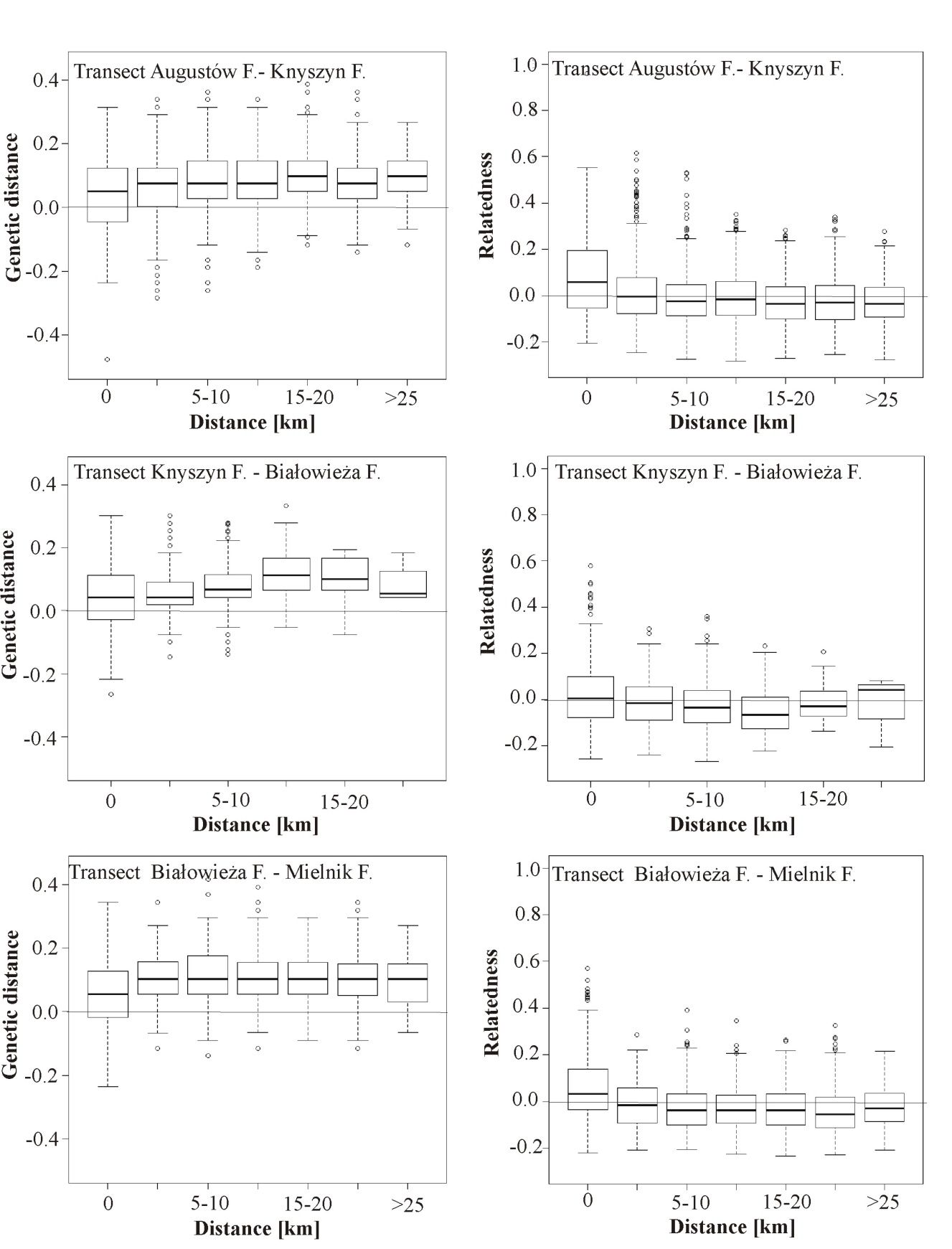


Fig. S5. Rousset’s individual genetic distance (a_r_) and Queller and Goodnight relatedness coefficient calculated in six distance classes for yellow-necked mice collected along three transects (TAK, TKB, TBM).
